# ﻿New and poorly known “araphid” diatom species (Bacillariophyta) from regions near Lake Titicaca, South America and a discussion on the continued use of morphological characters in “araphid” diatom taxonomy

**DOI:** 10.3897/phytokeys.187.73338

**Published:** 2021-12-13

**Authors:** Eduardo A. Morales, Carlos E. Wetzel, Luc Ector

**Affiliations:** 1 Water Laboratory, University of Évora, P.I.T.E. Rua da Barba Rala No. 1, 7005–345 Évora, Portugal; 2 Institute of Earth Sciences – ICT, University of Évora, Rua Romão Ramalho n°. 59, 7000–671 Évora, Portugal; 3 Observatory for Climate, Environment and Biodiversity (OCEB), Environmental Research and Innovation (ERIN) Department, Luxembourg Institute of Science and Technology (LIST), 41 rue du Brill, 4422 Belvaux, Luxembourg

**Keywords:** Andean mountains, fragilarioid diatoms, morphology, South America, traditional taxonomy

## Abstract

Based on two Andean Altiplano samples and on light and scanning electron microscopy analyses, we present six new species of “araphid” diatoms in the genus *Pseudostaurosira*, *P.aedes***sp. nov.**, *P.frankenae***sp. nov.**, *P.heteropolaris***sp. nov.**, *P.oblonga***sp. nov.**, *P.occulta***sp. nov.**, and *P.pulchra***sp. nov.** Additional data are provided for four other known taxa, *Nanofrustulumcataractarum*, *N.rarissimum*, *P.sajamaensis* and *P.vulpina*, the latter species corresponding to a stat. nov. based on a variety of *P.laucensis*. Each taxon is described morphologically and compared with closely related published taxa, using characters such as axial area, virgae, vimines, areolar shape, volae, internal striae depositions, spines, flaps and apical pore fields, which are not usually used for species distinction within the genus. It is our intention that the detailed morphological descriptions of each taxon and the elaborate comparative tables we provide serve as a basis for correction of neo and paleo-databases for the Altiplano to produce a better account of autecological data and ecological change in the region. Some arguments for our continued use of a morphologically based approach are given in the context of rapid environmental degradation in the Andes and the difficulties in applying molecular approaches in countries such as Bolivia.

## ﻿Introduction

In the last two decades, many new “araphid” taxa have been described, clarifying the morphological concepts of existing genera or better delimiting the boundaries of widely reported species (e.g. Lange-Bertalot and Ulrich 2014; Wetzel and Ector 2015; Wengrat et al. 2016; Almeida et al. 2017; García et al. 2017; Van de Vijver et al. 2020a). The study of type material helped in the latter endeavor, which coupled with illustrated reports and newly found populations, gave a clearer view of diagnostic characters and added other features that had not been used before for recognition of purportedly well-known species (e.g. Edlund et al. 2006; Cejudo-Figueiras et al. 2011; Wetzel et al. 2013a, b; Talgatti et al. 2014; Delgado et al. 2015; Van de Vijver et al. 2020a, b). Such is the case, for example, with *Pseudostaurosirabrevistriata* (Grunow) D.M. Williams & Round (Morales et al. 2015, 2019b) and *Staurosirellapinnata* (Ehrenberg) D.M. Williams & Round (Morales et al. 2013a, 2019a). The literature for both is extensive, revealing a history of taxonomic drift, lumping and imprecise reports of their autecology (Morales et al. 2013a, 2014c, 2019b).

These morphological studies continue to be important in the resolution of taxonomic issues and taxa delimitation. New morphological descriptions and taxonomic revisionary work provide a series of testable hypotheses that constitute the grounds upon which further progress can be made in fields such as systematics, ecology, conservation, etc. (de Carvalho et al. 2007; Haszprunar 2011).

Though molecular studies are becoming increasingly important in the resolution of taxonomical issues at the species level, both sources of information, morphological and molecular, ought not to be divorced and are rather complementary since morphological studies generate hypotheses based on the phenotype, while molecular studies do it based on the genotype. One dataset can be used as a confirmation of the other. The concatenation of both sources of information could produce a stronger and better-supported taxonomic system that can be translated, for example, into a practical tool to be used at the bench during routine identification analyses (Kahlert et al. 2019).

However, the colossal task that represents the production of fully operating barcode datasets (Zimmermann et al. 2014; Kelly et al. 2018) that are applicable to nature deems “traditional” morphological analyses a continued fast and viable way to produce data and hypotheses on taxa identities and distinctiveness. The same can be said for reliable phylogenies that aim to express natural classifications (see Li et al. 2018 and discussion in Morales et al. 2019b) but production of phylogenies is another matter, a different stage in the process of studying biodiversity that we are not concerned with in the present contribution. Here we deal only with a first stage of discovery, description of traits and a comparative analysis to justify the hypothetical placement of the treated taxa under given genera.

The study of “araphid” diatoms from high Andean ecosystems is important since they are frequent in current and paleoecological samples. Their abundance and distribution have been used to determine past climate, water level and precipitation changes, salinity and ionic composition, and temperature variations (e.g. Servant-Vildary 1978; Servant-Vildary and Roux 1990; Fritz et al. 2004; Tapia et al. 2006). But also from the taxonomic standpoint, it becomes relevant to describe species and produce inventories and autecological data for Andean “araphids” and other diatoms, especially because mountainous areas are affected more rapidly by climate change than any other land ecosystems (Marengo et al. 2011; Cuesta et al. 2012; Michelutti et al. 2015). Coupled with land use effects, threats to ecological stability with the anticipated negative effects on human and other populations are beginning to be observed in these areas (Suárez et al. 2011). A thorough knowledge of the taxonomy and autecology of diatoms could provide an aid in the conservation of Andean areas and their communities.

The examination of recent samples from Bolivia and Argentina has shown that the Andes contains hot spots for “araphid” species diversity (e.g. Morales et al. 2012b, 2019b; Grana et al. 2018; Seeligmann et al. 2018; Guerrero et al. 2019). Study of these sites could facilitate the taxonomic clarification of several known taxa, quickly produce new species and generate inventories that can then be applied to other Andean regions.

The present paper aims to continue the morphological description of diatom taxa present in the region contiguous to Lake Titicaca, concretely in the Desaguadero River and adjacent zones. This area is affected by natural soil erosion, typical of the Bolivian Altiplano, but also by land use and water use changes that have been affecting the area for several decades (UNEP-United Nations Environmental Program 1996). The recent international news about the drying of neighboring Lake Poopó, the site where a substantial amount of fauna and flora thrived and from where human groups, descendants of the millenary Urus tribes, have been displaced (Richard and Contreras 2015), furnished evidence of the urgency of basic studies such as the present one. The long-term goal is to improve the paleolimnological/paleoclimatic characterizations conducted in the area (e.g. Servant-Vildary 1978; Servant-Vildary and Blanco 1984; Tapia et al. 2003) and to provide baseline data for conservation and management practices in the region.

Concretely, we present six new species together with comparative analyses with published morphologically closely related taxa, and additional morphological information and comparative data for other four species described from the Andes and elsewhere. For all ten taxa, a pertinent discussion is presented to aid in their distinction and identification.

## ﻿Methods

The study area, the southern region contiguous to Lake Titicaca, was already described in a geographical and ecological context by Morales et al. (2012b, p. 42–44) and Grana et al. (2015, table 1, fig. 4 and p. 815). Epipsammon material used in the present study is the same as that described in Grana et al. (2015), collected from rivers Desaguadero and Sajama with the aid of a turkey baster and fixed with 20 drops of 40% formaldehyde in the field.

For LM analysis, subsamples of 20–30 mL were mixed with a similar volume of 70% HCl. The mixture was boiled for 45 min and rinsed 8 times using distilled H_2_O. Drops of cleaned slurry were dried on coverslips overnight at room temperature. Permanent slides were mounted using the synthetic medium Naphrax. Slides were analyzed using a Zeiss Universal microscope equipped with differential interference contrast optics, a 1.25 optivar, and a Plan 100X, 1.25 NA, immersion objective. Images were taken using a Jenoptik CF color digital camera and ProGres CapturePro ver. 2.8 software.

For SEM analysis, about 10 to 20 mL aliquots of raw samples were digested with concentrated H_2_O_2_ and heated for 24 h using a sand bath. Then, samples were allowed to cool and settle (ca. 1 cm/h) and 80 to 90% of supernatant was eliminated by vacuum aspiration. A volume of 1 mL of HCl acid (37%) was added and the preparation was allowed to settle for 2 h. Subsequently, the sample was rinsed and decanted three times using deionized water. Approximately 100 mL aliquots of clean material were filtered and rinsed with deionized water through glass fiber filters with a 3 μm pore diameter. Coating with platinum was accomplished using a BAL-TEC MED 020 Modular High Vacuum Coating System for 30 s at 100 mA. A Hitachi SU-70 electron microscope operated at 5 kV and 10 mm distance was used for SEM analysis. Micrographs were digitally manipulated and plates containing LM and SEM pictures were mounted using Photoshop CS3.

Identification of taxa was performed using literature published for South America (Metzeltin and Lange-Bertalot 1998, 2007; Rumrich et al. 2000; Metzeltin et al. 2005). Taxonomic articles by Frenguelli (1939), Patrick (1961), Manguin (1964), Hohn (1966), Servant-Vildary (1986), Morales and Vis (2007) and Morales et al. (2007) were also used. Additionally, non-South American references were used such as the general floras of Patrick and Reimer (1966, 1975), Krammer and Lange-Bertalot (1986, 1988, 1991a, b), Simonsen (1987), Lange-Bertalot (1993), Hofmann et al. (2011), and Lange-Bertalot et al. (2017), as well as references specialized in certain genera such as *Navicula* (Lange-Bertalot 2001), *Pinnularia* (Krammer 2000) and cymbelloids (Krammer 1997a, b). Descriptions of the new taxa are based on measurements of 30 valves and observation of more than 100 individuals of each taxon under both LM and SEM. Morphological terminology follows Anonymous (1975), Ross et al. (1979) [both references used for terminology applied to striae, areolae and spines], Barber and Haworth (1981) [for terminology related to valve shape and striae orientation], Williams and Round (1987) and Round et al. (1990) [both references used for terminology on areolar substructures, girdle band features and apical pore field characteristics].

## ﻿Results

### ﻿ Nanofrustulum cataractarum

Taxon classificationPlantaeFragilarialesStaurosiraceae

(Hustedt) C.E. Wetzel, E. Morales & Ector in Morales et al. 2019b, Plant Ecology and Evolution 152, p. 275.

5251216B-AC50-5692-B5DD-59CE1B59CE26

**Figure 1.**
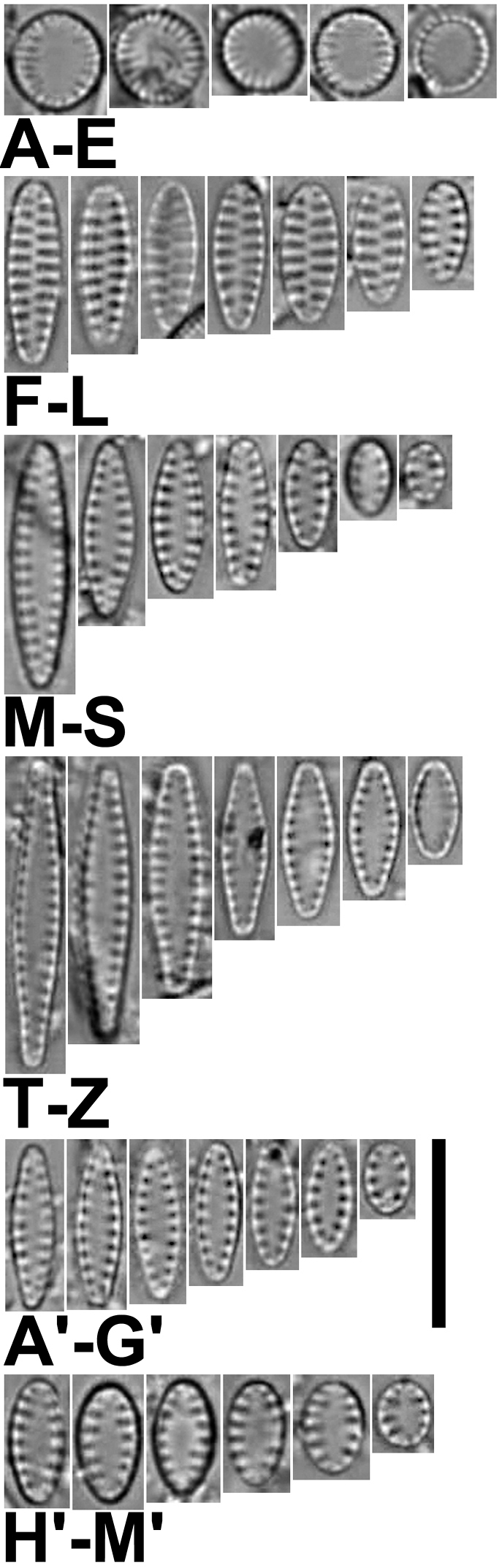
**A–M**’ LM images of little known and new “araphid” diatoms from the Bolivian Altiplano **A–E***Nanofrustulumcataractarum***F–L***N.rarissimum***M–S***Pseudostaurosirasajamaensis***T–Z***P.pulchra* sp. nov. (Fig. 1U is the holotype) **A’–G**’ *P.aedes* sp. nov. (Fig. 1A’ is the holotype). **H’–M**’ *P.heteropolaris* sp. nov. (Fig. 1I’ is the holotype). Scale bar: 10 µm.

Figs 1A–E (LM), 2A, B (SEM)

#### Basionym.

*Melosiracataractarum*Hustedt 1938, Archiv für Hydrobiologie, Supplement 15, p. 142, pl. 9, figs 6–7.

Most current illustrations of type material: Wetzel et al. 2013a, figs 1A–AB, 2A–G; Beauger et al. 2019, figs 93, 94.

#### Synonym.

*Pseudostaurosiracataractarum* (Hustedt) C.E. Wetzel, E. Morales & Ector in Wetzel et al. 2013a Acta Nova 6(1–2), p. 60.

#### Comment.

The taxon was first described for insular Asia, specifically from Java, Indonesia, by Hustedt (1938). Type material was reanalyzed by Wetzel et al. (2013a) and Beauger et al. (2019) and regional and worldwide distributions were presented in Wetzel et al. (2013a) and Grana et al. (2015).

**Figure 2.**
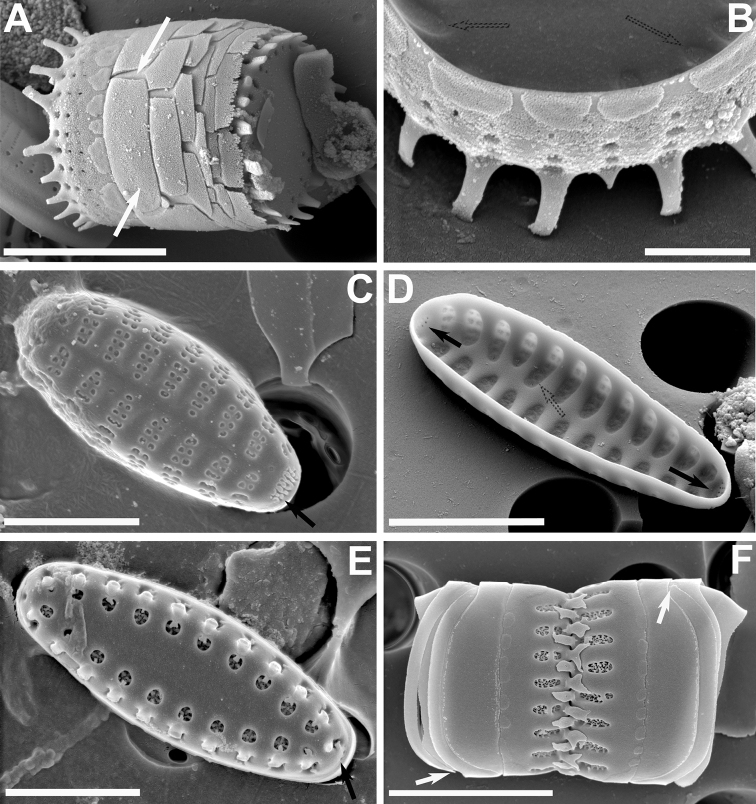
**A–F** SEM images of little known “araphid” diatoms from the Bolivian Altiplano **A, B***Nanofrustulumcataractarum***A** specimen from the Desaguadero River, showing quasifract girdle elements with prominent ligulae (white arrows) **B** specimen from the Sajama River showing common internal depression into which the areolae open (dotted arrows) and the blister-like depositions of silica at the abvalvar edge of the mantle **C, D***N.rarissimum* from the Desaguadero River **C** small, spineless valve **D** internal view showing apical and foot pole pore fields (black arrows) and internal depressions containing all areolae within a stria (dotted arrow) **E, F***Pseudostaurosirasajamaensis* from the Desaguadero River **E** top view showing gradual transition of valve face to mantle and the reduced apical pore fields (black arrow) **F** side view of two half cells still attached by heavily silicified spines. Notice open girdle elements (white arrows). Scale bars: 1 µm (**B**); 3 µm (**C, E**); 4 µm (**A, D**); 5 µm (**F**).

As presented in Table 2 in Grana et al. (2015), *N.cataractarum* from Bolivia (Figs 1A–E, 2A, B) are smaller (length and width: 4.5–5 µm) than specimens in Asian type material (length 5.8–8.2, width 5.4–7.2), and the stria density of the Bolivian population is higher than that from Asia (18–20 and 15–28 in 10 µm, respectively). Regarding the areola density there is a complete overlap between both populations (2.5–3.5 in Bolivian specimens and 1–4 per 1 µm in Asian ones). Other features, such as the pattern of areolation in both valve face and mantle, the ample, round to oval axial area, the round to slightly elongated base and flattened body of the spines with small lateral projections, are similar in both populations. Also, the depression into which the areolae from valve face and mantle open internally is similar in Bolivian and Asian specimens (Fig. 2B). The features of the girdle elements with short but wide body and prominent ligula is also comparable in both populations. The Bolivian specimens tended to have more prominent blister depositions at the abvalvar edge of the mantle (Fig. 2B). All populations reported from around the world lack apical pore fields, and areolae flaps or spine stipules have not been reported either.

Taking into account all the above-mentioned reports, the dimensions for this taxon are length: 2.8–8.2 µm; width: 2.7–7.2; stria density: 15–29 in 10 µm; areola density: 1–4 in 10 µm.

In Bolivia, the taxon has been found in the Desaguadero and Sajama rivers. Fig. 2A is the first illustration of the taxon for the Desaguadero River.

### ﻿ Nanofrustulum rarissimum

Taxon classificationPlantaeFragilarialesStaurosiraceae

E. Morales, Novais, C.E. Wetzel & Ector in Morales et al. 2019b, Plant Ecology and Evolution 152, p. 269, figs 1A–K, 2A–D.

21B12650-844D-543E-904D-DB958A59E4BE

Figs 1F–L (LM); 2C, D (SEM)

#### Comment.

This taxon was first described by Morales et al. (2019b) from the Desaguadero River. Here we present illustrations of specimens from the Sajama River for the first time (Fig. 1F–L). Thus far, this diatom has only been seen in samples from these two sites.

The specimens found in the Sajama sample fit the dimensions of the type population, except for the length, with Sajama River specimens being shorter (5.1–9.7 µm). At the SEM levels, no differences were noted between specimens from both sites.

Our reanalysis of Desaguadero River material yielded small valves that are spineless (Fig. 2C), but that had all the other features similar to those of larger, spiny specimens. Also, we were able to capture the apical and foot pole pore fields from an internal view (Fig. 2D), confirming that both are developed, but that the one at the foot pole is larger. Additionally, we were able to confirm the raised nature of the axial area and virga in internal view (Fig. 2D), which leaves all the areolae within a stria open into a single internal depression.

The smaller specimens found in the Sajama River sample expand the length range of this taxon which now has the following diagnostic measurements length: 5.5–9.5; width 2.5–3.3; stria density 12–13 in 10 µm.

### ﻿ Pseudostaurosira sajamaensis

Taxon classificationPlantaeFragilarialesStaurosiraceae

E. Morales & Ector in Morales et al. 2012b, Fottea 12, p. 45, figs 12–26, 45–56.

EACB6B1E-3CE3-5DC2-AFDB-3299DE1D43C0

Figs 1M–S (LM); 2E, F (SEM)

#### Comment.

This taxon was first described from the Desaguadero River; here we also report its finding in the Sajama River. The population found in the latter falls well within the features described by Morales et al. (2012b) based on the Desaguadero River sample.

At the LM level, the narrowly elliptical valves with pointy ends and coarser striation can be used to recognize the taxon in a first instance. At the SEM level, the transapically elongated and wide areolae (Fig. 2F) are present in the majority of specimens from both sites reported here and the valve face typically and gradually transitions into the mantle, making the striae on the mantle partially visible in top outer views (Fig. 2E, and also see LM images in Figs 1M–S). The areolae vary in shape from round to trapezoid on the valve face and there is usually one very large trapezoid areola on the mantle. The volae are conspicuous and form an entangled structure. The spines have a flattened body, but they look sagittate in lateral view due to the presence of well-developed stipules. These spines sometimes have a V-shaped cleft on its back, and the tips terminate in a single or two ends (diapason-shaped) that have serrate borders pointing downward. The stipules are well-developed giving the spines a profile resembling an arrow (sagittate).

As was the case with the Desaguadero population, the Sajama River specimens lack or have weakly developed apical pore fields. Regarding the girdle elements, the valvocopula is conspicuously wider than the rest of the elements and all are open (Fig. 2F).

No changes in valve diagnostic measurements were yielded by our observations of Sajama River material.

### ﻿ Pseudostaurosira pulchra

Taxon classificationPlantaeFragilarialesStaurosiraceae

E. Morales, C.E. Wetzel & Ector sp. nov.

9FAC2BE6-9DE8-5294-8814-17E3B6C72CDE

Figs 1T–Z (LM), 3A–F (SEM)

#### Holotype.

Slide ANSP GC 26815, Fig. 1U. Diatom Herbarium, Academy of Natural Sciences, Philadelphia (ANSP). **Isotype.** Slide DBOL-0246a, Diatomotheca Boliviensis (before HCUCB), Cochabamba, Bolivia.

#### Type locality.

Bolivia. Sajama Province, Department of Oruro, Desaguadero River, epipsammon, 17°23'51"S; 68°14'33"W, 3701 m elev., *leg.* G. Chávez, 05.07.2009.

#### Description.

**Figure 3.**
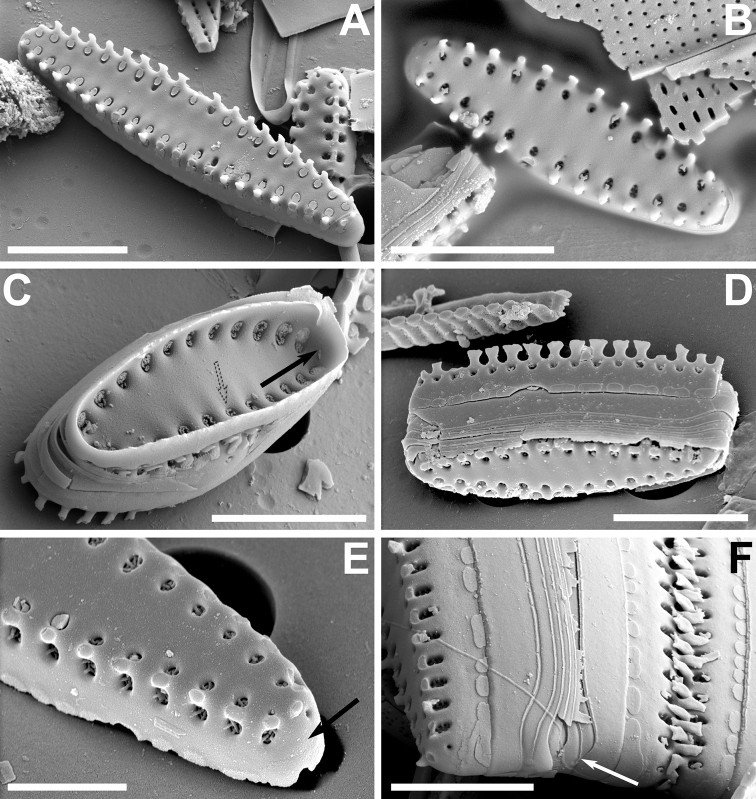
**A–F** SEM micrographs of *Pseudostaurosirapulchra* sp. nov. from the Bolivian Altiplano **A, B** outer views of valves showing axial area, striae and spines features **C** inner view of valve still attached to neighboring cell. Black arrow indicates absence of apical pore field. Dotted arrow points to depression into which the stria opens internally **D** tilted view of frustule. Notice larger valvocopula, lateral growth of spines and blister-like depositions on abvalvar edge of mantle **E** outer view of valve tip. Black arrow denotes absence of apical pore field. Notice round to elliptical spine base and larger areola on valve mantle. **F** frustule attached to neighboring valve by means of heavily silicified spines. Notice open girdle elements (white arrow) and depositions along abvalvar mantle edge. Scale bars: 2 µm (**E**); 4 µm (**C, F**); 5 µm (**A, B, D**).

Frustules rectangular in girdle view (Fig. 3D), joined together by interlocking spines (Figs 3C, F). Valves narrowly lanceolate, isopolar, with abrupt transition from valve face to mantle. Rostrate valve ends in larger specimens, broadly rounded in smaller ones (Figs 1T–Z). Axial area narrowly lanceolate (Figs 1T–Z, 3A, B) and externally and internally depressed with respect to virgae (Fig. 3A, B). Internally, striae open in small trapezoid, transapically elongated depressions (Fig. 3C). Vimines short and wide, restricted to the valve face/mantle junction; additional ones rarely present on either valve face or valve mantle (Fig. 3C). Striae typically composed by two narrow, round to elliptic areolae, one on valve face and a larger one on the valve mantle (Figs 3E, F). Well-developed volae, arising from the areolar inner periphery and projecting inwards forming a loose mesh-like structure (Figs 3C, E). Flaps usually present in various stages of development, typically single and disk-like on valve face and two or more on mantle areolae (Fig. 3A). Spines originating from vimines at the valve face/mantle junction; solid, with round to elliptical base, wider that the vimines they sit on; flattened, with biconcave sides and spatulate body, truncated (cut) at the top or with a short bifurcation (Fig. 3A, B, D, E, F). Stipules absent. Apical pore fields absent (Fig. 3C, E). Well-developed blister-like depositions present on abvalvar edge of mantle also covering both apices (Fig. 3D–F). Girdle elements variable in number, open, lacking pores, ligulated, with larger valvocopula (Fig. 3D, F).

Dimensions (n > 50): Length 5–22 μm; width 2.4–3.0 μm; striae 13–16 in 10 μm.

#### Etymology.

The epithet makes reference to the neat and eye-catching morphology of the frustules.

#### Distribution.

Found in the Desaguadero and Sajama rivers.

### ﻿ Pseudostaurosira aedes

Taxon classificationPlantaeFragilarialesStaurosiraceae

E. Morales, C.E. Wetzel & Ector sp. nov.

02C317AB-EE8E-57C3-AB16-D70CA6F527A6

Figs 1A ’–G’ (LM), 4A–F (SEM)

#### Holotype.

Slide ANSP GC 26815, Fig. 1A’, Diatom Herbarium, Academy of Natural Sciences, Philadelphia (ANSP). **Isotype.** Slide DBOL-0246a, Diatomotheca Boliviensis (before HCUCB), Cochabamba, Bolivia.

#### Type locality.

Bolivia. Sajama Province, Department of Oruro, Desaguadero River, epipsammon, 17°23'51"S; 68°14'33"W, 3701 m elev., *leg.* G. Chávez, 05.07.2009.

#### Description.

**Figure 4.**
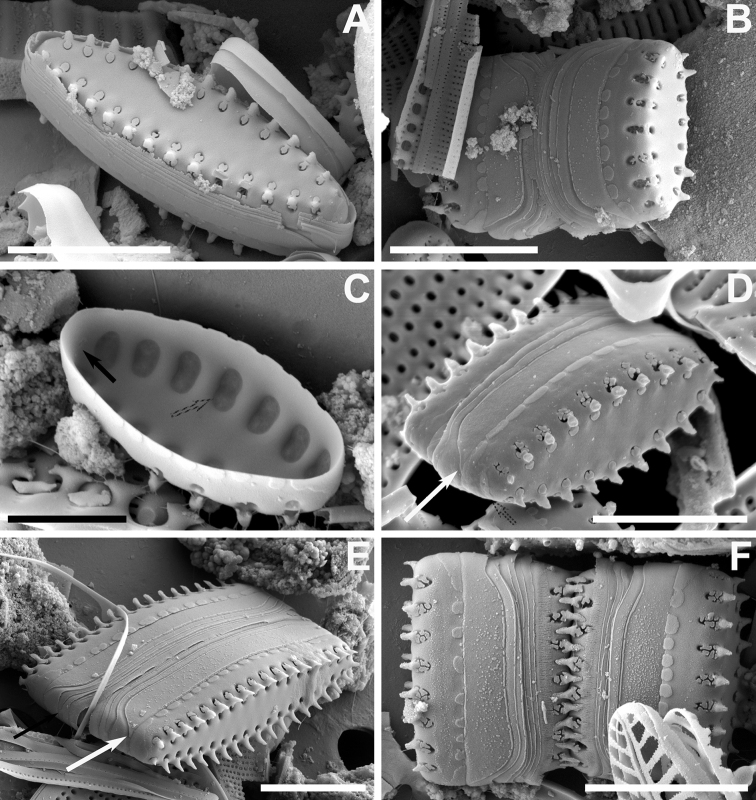
**A–F** SEM images of *Pseudostaurosiraaedes* sp. nov. from the Bolivian Altiplano **A** top, tilted view of valve showing axial area, slightly raised virga, valve face areolae covered with bilobed or disk-like flaps, and slightly larger valve mantle areola covered with two or more flaps **B, D, E, F** girdle views showing features of the valve mantle, open girdle elements with larger valvocopula (white arrows in **D** and **E**). Notice serrate spines with well-developed stipules in **F** which have varying patterns **C** inner view showing reduced apical pore field (black arrow) and single depression into which the areolae open internally (dotted arrow). Scale bars: 4 µm (**D**); 5 µm (**A–C, E, F**).

Frustules rectangular in girdle view (Fig. 4B, D–F), joined together by interlocking spines (Fig. 4F). Valves narrowly elliptic with rounded ends, isopolar, with abrupt transition from valve face to mantle (Fig. 1A’–G’). Axial area narrowly lanceolate (Figs 1A’–G’, 4A, B, D), externally only slightly below the virga (Fig. 3A, D, E). Internally, axial area and virgae raised, leaving the striae in large elliptic or 8-shaped, transapically elongated depressions (Fig. 4C). Vimines shorter than virgae and wide, restricted to the valve face/mantle junction; additional ones rarely present on valve mantle (Fig. 4B). Striae typically composed by two narrow, elliptic to trapezoid areolae, one on valve face and a slightly larger one on the valve mantle (Fig. 4A, B, D, E). Volae arising from the areolar inner periphery and projecting inwards forming a tightly packed mesh-like structure (Fig. 4B, C). Flaps frequently present in various stages of development, typically one disk-like or bilobate on valve face and two or more of different shape on valve mantle areola (Fig. 4A, B, D–F). Spines originating from vimines at the valve face/mantle junction, solid, with elliptic to rectangular base, as wide as the vimines; conical body with a roughly triangular profile and serrate, pointy tips. Spines have a general arrowhead-like appearance when seen form their posterior ends. (Fig. 4A–F). Stipules well-developed giving spines a sagittate shape and having themselves varying shapes in girdle view (Fig. 4D–F). Apical pore fields very reduced with no more than 3 round poroids, usually externally obliterated by an apical blister (Fig. 4C, D, E). Well-developed blister-like depositions present on abvalvar edge of mantle (Fig. 4B–F). Girdle elements variable in number, open, lacking pores, ligulated, with larger valvocopula (Fig. 4B, D–F).

Dimensions (n > 50): Length 2.9–12.3 μm; width 2.1–2.3 μm; striae 15 in 10 μm.

#### Etymology.

The species epithet makes reference to the difficulty in the LM distinction of this diatom from co-occurring species with similar outline.

#### Distribution.

Found in the Desaguadero River.

### ﻿ Pseudostaurosira heteropolaris

Taxon classificationPlantaeFragilarialesStaurosiraceae

E. Morales, C.E. Wetzel & Ector sp. nov.

0BA89859-7D0C-5335-86DE-5365D3F97D5C

Figs 1H ’–M’ (LM), 5A–F (SEM)

#### Holotype.

Slide ANSP GC 26815, Fig. 1I’, Diatom Herbarium, Academy of Natural Sciences, Philadelphia (ANSP). **Isotype.** Slide DBOL-0246a, Diatomotheca Boliviensis (before HCUCB), Cochabamba, Bolivia.

#### Type locality.

Bolivia. Sajama Province, Department of Oruro, Desaguadero River, epipsammon, 17°23'51"S; 68°14'33"W, 3701 m elev., *leg.* G. Chávez, 05.07.2009.

#### Description.

**Figure 5.**
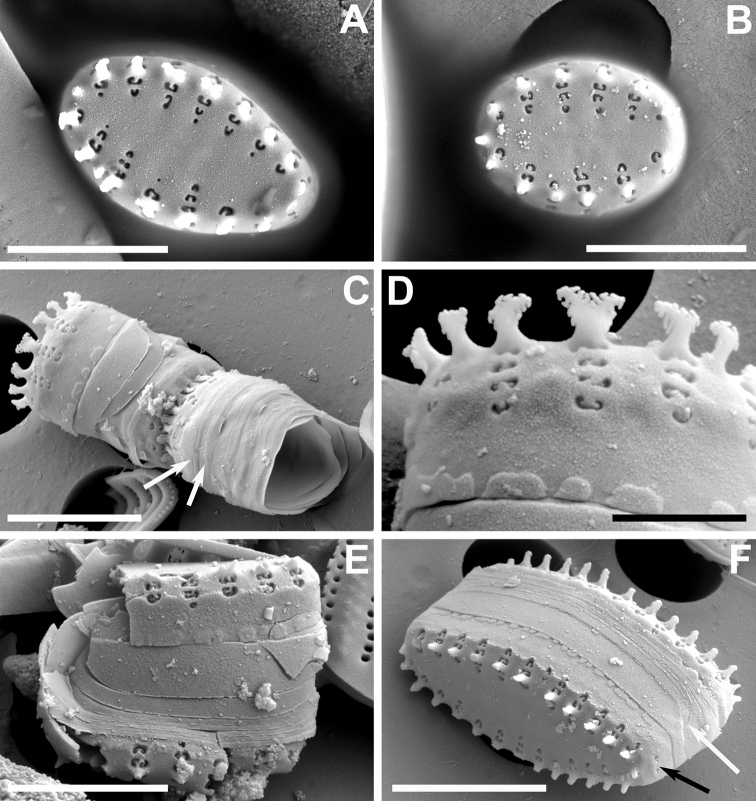
**A–F** SEM images of *Pseudostaurosiraheteropolaris* sp. nov. from the Bolivian Altiplano **A, B** Valve views showing striation pattern and features of the axial area, virgae, vimines, and spine location **C** side view of complete frustule and neighboring cell showing girdle bands (white arrows denote open copulae). White arrows point to open girdle elements **D** close up of **C** showing details of spines with bifurcations with pinnatifid projections, characteristics of the striae on valve mantle and the features of the blisters **E** broken frustule with girdle bands. Pattern of volae within areolae is also shown **F** frustule in side, tilted view. Notice open copulae (white arrow) and reduced apical pore field with cavernous poroids (black arrow). Scale bars: 1 µm (**D**); 3 µm (**A–C, E**); 4 µm (**F**).

Frustules rectangular in girdle view (Fig. 5C, D), joined together by interlocking spines (Fig. 5C). Valves ovoid to elliptic, heteropolar, with gradual transition from valve face to mantle (Figs 1H’–M’, 5A–F). Axial area elliptic (Figs 1H’–M’, 5A, B, F), externally slightly depressed with respect to virgae, internally at the same level as virgae (Fig. 5A, D, E). Virgae much wider than striae (Fig. 5A, D–F). Vimines shorter than virgae and wide (Fig. 5A, B, D–F). Striae composed of narrow, apically elongated, rectangular to semi-elliptic areolae (Fig. 5A–F). Areolae diminish in size from valve face/mantle junction towards striae extremes at about the same rate (Fig. 5F). Volae arising from up to two points (typically one) within the areolar inner periphery, projecting inwards (Fig. 5A, B, D–F). Base of volae thick and giving areolae a C-shape (Fig. 5A, B). Flaps absent. Spines originating from vimines at the valve face/mantle junction, solid, with elliptic to rectangular base, wider than the vimines they sit on; cylindrical body with biconcave sides, spatulate tips with pinnatifid (with deep lateral) bifurcations (Fig. 5C, D). Stipules absent (Fig. 5D). Apical pore fields very reduced with no more than 3 cavernous poroids in external view; not seen in internal view (Fig. 5F). Small blister-like depositions present on abvalvar edge of mantle, including at the valve apices (Fig. 5C–F). Girdle elements variable in number, open, lacking pores, ligulated, with larger valvocopula (Fig. 5C, E, F).

Dimensions (n > 50): Length 3.0–4.3 μm; width 2.6–3.3 μm; striae 13–16 in 10 μm.

#### Etymology.

The epithet of this species refers to its typical heteropolar valve outline.

#### Distribution.

Found in the Desaguadero River.

### ﻿ Pseudostaurosira vulpina

Taxon classificationPlantaeFragilarialesStaurosiraceae

(Lange-Bertalot & U. Rumrich) E. Morales stat. nov.

FAFB9B1F-C73C-566E-8E01-451E48DD2231

**Figure 6.**
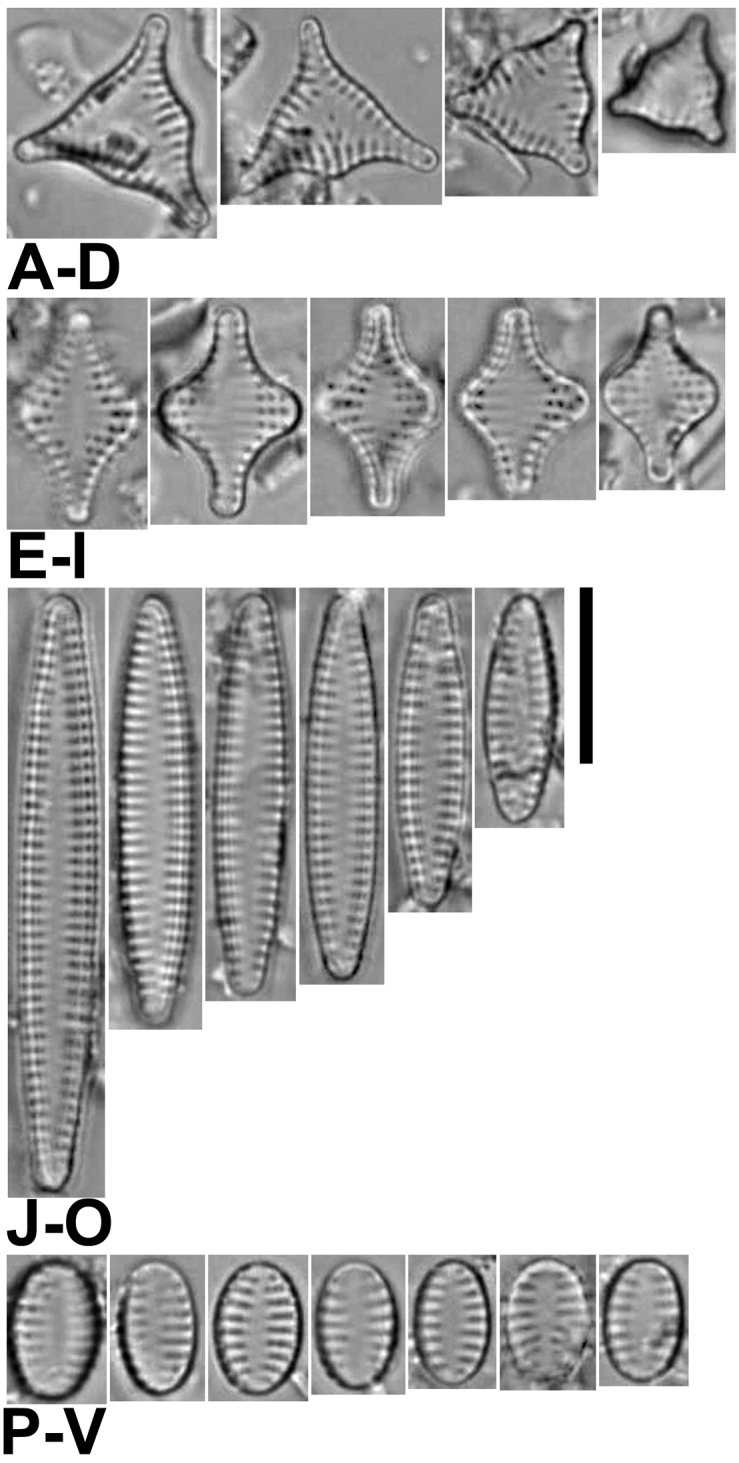
**A–V** LM images of little known and new “araphid” diatoms from the Bolivian Altiplano **A–D***Pseudostaurosiravulpina* stat. nov. **E–I***P.frankenae* sp. nov. (Fig. 6E is the holotype) **J–O***P.occulta* sp. nov. (Fig. 6K corresponds to the holotype) **P–V***P.oblonga* sp. nov. (Fig. 6R corresponds to the holotype). Scale bar: 10 µm.

Figs 6A–D (LM), 7A–F (SEM)

#### Basionym.

Staurosiralaucensisvar.vulpina Lange-Bertalot & U. Rumrich *in*Rumrich et al. 2000, Diatoms of the Andes from Venezuela to Patagonia/Tierra Del Fuego, Iconographia Diatomologica 9, p. 223–224, Plate 10, Figs 1–11.

#### Comment.

This taxon was first described from the Chilean Altiplano and was found mixed with the nominate variety *Pseudostaurosiralaucensis* (Lange-Bertalot & Rumrich) E. Morales & Vis (in Rumrich et al. 2000, p. 222, figs 10–20, 22, 23; Morales and Vis 2007, p. 25). This was the probable reason why Lange-Bertalot and Rumrich (in Rumrich et al. 2000) decided to describe it as a variety. However, we found the var.vulpina isolated from the nominate variety in the Desaguadero River sample. This population, like the one reported from Chile, exhibits a range of sizes which is probably showing that it is undergoing asexual reproduction and its size is most probably being re-established through sexual reproduction.

**Figure 7.**
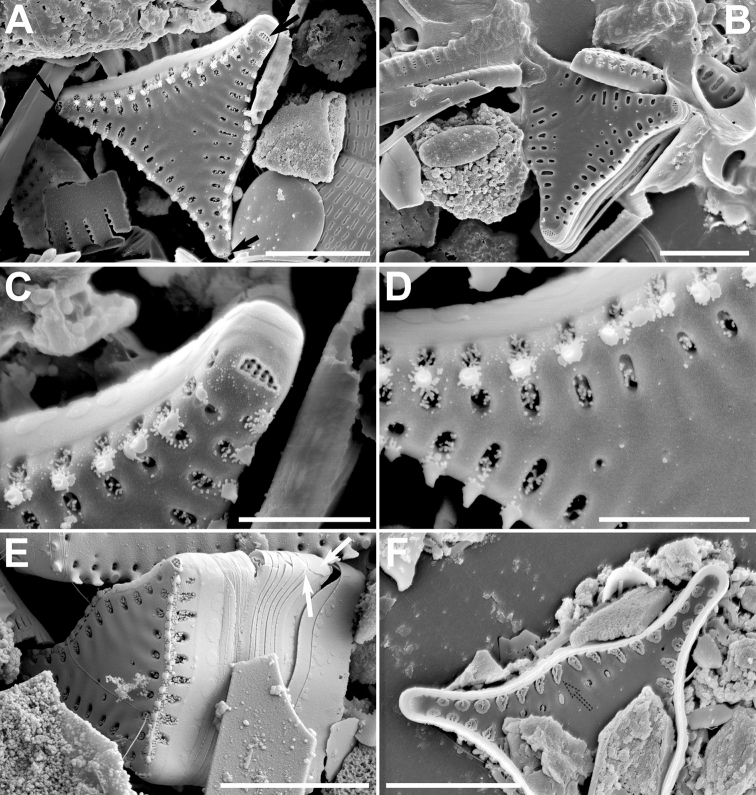
**A–F** SEM images of *Pseudostaurosiravulpina* sp. nov. **A, B** valve views showing striation pattern, features of the axial area, spine position and presence of depressed apical pore fields (black arrows) **C, D** close-ups with details of apical pore fields areolae and spines **E** tilted view of a frustule showing open girdle bands with overlapping extremes (white arrows) **F** internal view showing apical pore fields and the internal disk-like areolar depositions. Scale bars: 2 µm (**C, D**); 5 µm (**A, B, E, F**).

At the LM level, this taxon is distinguished by its typical triradiate shape (Fig. 6A–D). Between each of the arms there is also a central inflation that becomes more pronounced as the valve decreases in size (Fig. 6C, D). At the SEM level, the axial area is depressed in external view with respect to the virgae, while internally it is at the same level as the latter. Each of the arms has an apical pore field that lies within a shallow, irregular depression (Fig. 7A–C) and opens to the valve interior as a plain plate of pores (Fig. 7F). The transapically elongate areolae bear well-developed volae (Fig. 7A–E), which allow inorganic deposition of an inverted cone-like structure internally covering the areolae, sometimes filled with extra depositions in their hollow interior (Fig. 7F). The spines are conical, but also it is common to find them as incipient, shapeless spines that are generated from the virgae and the vimines (Fig. 7E). The girdle elements vary in number, lack perforations and all are open (Fig. 7E). The valvocopula is wider. At the open side, each element has its terminations superimposing each other (Fig. 7E).

Dimensions (n > 10): Length (from the extreme of one arm to the other) 4.8–13.0 μm; width (from one swollen central area to its opposite side) 4.1–5.6 μm; stria density (measured from arm to arm) 14–16 in 10 μm. The dimensions are given here for the first time since the original description in Rumrich et al. (2000) did not include them. Table 3 contains additional characteristics that are used below for comparative purposes in Discussion.

### ﻿ Pseudostaurosira frankenae

Taxon classificationPlantaeFragilarialesStaurosiraceae

E. Morales, C.E. Wetzel & Ector sp. nov.

0A997D42-E50A-5F0B-9614-7EEB3F863E08

Figs 6E–I (LM), 8A–F (SEM)

#### Holotype.

Slide ANSP GC 26815, Fig. 6E, Diatom Herbarium, Academy of Natural Sciences, Philadelphia (ANSP). **Isotype.** Slide DBOL-0246a, Diatomotheca Boliviensis (before HCUCB), Cochabamba, Bolivia.

#### Type locality.

Bolivia. Sajama Province, Department of Oruro, Desaguadero River, epipsammon, 17°23'51"S; 68°14'33"W, 3701 m elev., *leg.* G. Chávez, 05.07.2009.

#### Description.

**Figure 8.**
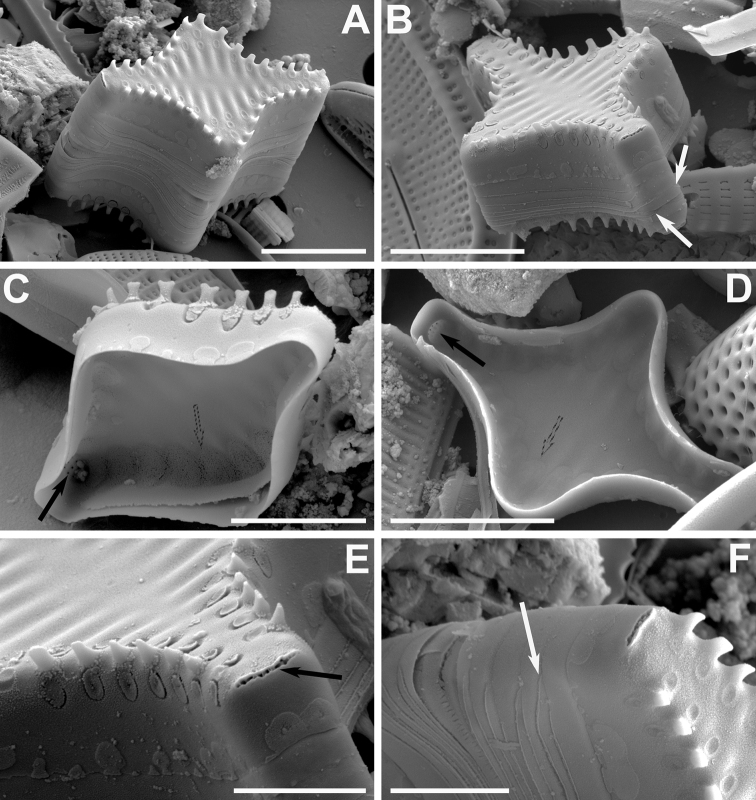
**A–F** SEM images of *Pseudostaurosirafrankenae* sp. nov. **A, B** titled frustules showing external features; notice open girdle elements in B (white arrows) **C, D** internal view of valves showing internal elliptic depositions on striae (dashed arrows) and depression of the apical pore field (black arrows) **E** close-up of frustule apex (**B**) showing the externally occluded apical pore field, showing only a single row of poroids (black arrow) **F** close-up of frustule tip (**A**) showing open girdle bands (white arrow). Scale bars: 2 µm (**E, F**); 3 µm (**C**); 5 µm (**A, B, D**).

Frustules rectangular with a curved middle portion in girdle view, joined together by interlocking spines. Valves cruciform, isopolar, with abrupt transition from valve face to mantle. Broadly rounded valve ends (Fig. 6E–I). Axial area lanceolate with a broad central inflation (Fig. 6E–I), externally and internally depressed with respect to virgae (Fig. 8A–C). Vimines short and wide (Fig. 8A, B, E, F). Striae typically composed round to elliptic areolae, decreasing in size towards the axial area (Fig. 8A, B); a single elliptical areola present on valve mantle (Fig. 8A–C, E, F). Well-developed volae, arising from the areolar inner periphery and projecting inwards (not shown here). Internally, depositions on volae forming round to elliptic structures, sealing areolae (Fig. 8C, D). Flaps persistent, a single disk-like one covering each areola in external view (Fig. 8A, B, E, F), 1–3 in enlarged mantle areolae (Fig. 8C). Spines originating from vimines at the valve face/mantle junction; solid, with round to elliptical base (Fig. 8E), wider that the vimines they sit on (Fig. 8F); flattened, with shallow biconcave sides, triangular in side view (Fig. 8A, B, E, F), and with spatulate body, bifurcate at the top (Fig. 8C). Stipules absent. Apical pore fields of cavernous appearance in external view, occluded by heavy silica deposition to the point only one row of pores can be seen (Fig. 8A, B, E, F). Internally, apical pore field opening into roundish depression, revealing several rows of round poroids (Fig. 8C, D). Well-developed blister-like depositions present on abvalvar edge of mantle also covering both apices (Fig. 8A–C, D–F). Girdle elements variable in number, open, lacking pores, ligulated, with larger valvocopula (Fig. 8A, B, F).

Dimensions (n > 30): Length 8.7–12.0 μm; width 6.7–7.7 μm; striae 14 in 10 μm.

#### Etymology.

The species is dedicated to the late Dr. Margot Franken, Professor and Researcher from the Ecology Institute, University Mayor de San Andrés, La Paz, Bolivia. Dr. Franken, originally from Germany, worked in Bolivia from 1985 to 2021, focusing on bioindication, urban ecology, water management and ecological architecture.

#### Distribution.

Found only in the Desaguadero River.

### ﻿ Pseudostaurosira occulta

Taxon classificationPlantaeFragilarialesStaurosiraceae

E. Morales, C.E. Wetzel & Ector sp. nov.

C499B059-A1A2-5442-872F-AA446F6CBEEA

Figs 6J–O (LM), 9A–F (SEM)

#### Holotype.

Slide BR-4679, Fig. 6K, Meise Botanic Garden, Belgium. **Isotype.** Slide DBOL-0249a, Diatomotheca Boliviensis (before HCUCB), Cochabamba, Bolivia.

#### Type locality.

Bolivia. Sajama Province, Department of Oruro, Sajama River, epipsammon, 17°30'33"S; 68°20'35"W, 4000 m elev., *leg.* G. Chávez, 05.07.2009.

#### Description.

**Figure 9.**
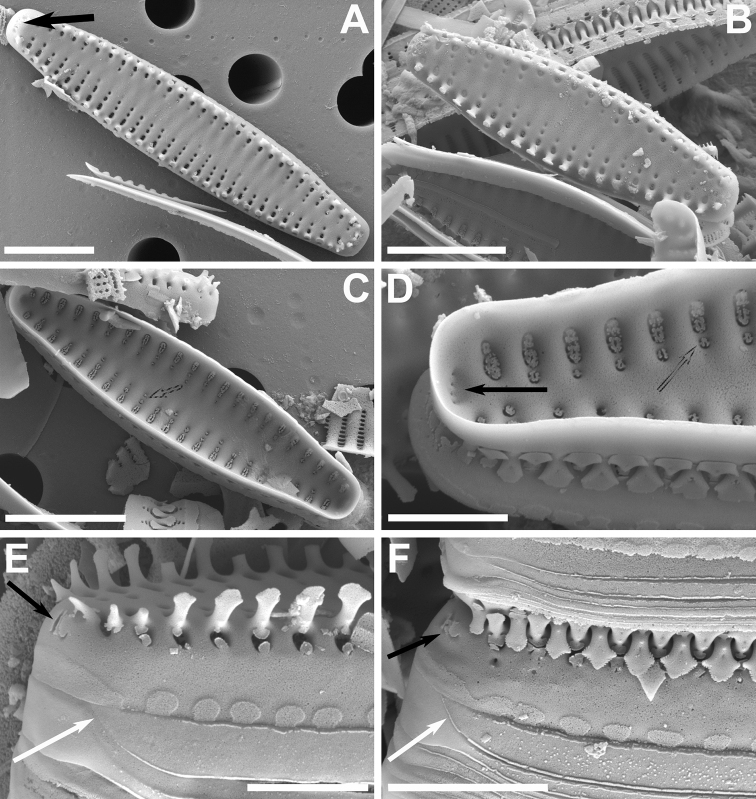
**A–F** SEM images of *Pseudostaurosiraocculta* sp. nov. **A, B** external views of valves showing apical pore fields (black arrow in A) and other features **C** internal view of valve showing depressions containing the striae (dashed arrow) **D** internal close-up showing apical pore field depression (black arrow) and striae depression (dashed line) **E** close-up on valve apex showing flaps on apical pore field (black arrow) and open girdle element (white arrow) **F** close-up on cell-cell connection showing apical pore field covered with flaps (black arrow) and open girdle element (white arrow). Scale bars: 2 µm (**D, E**); 3 µm (**F**); 5 µm (**A–C**).

Frustules rectangular in girdle view, joined together by interlocking spines. Valves lanceolate, isopolar with semi-gradual transition from valve face to mantle. Valve apices subrostrate with broadly rounded, somewhat squarish ends (Figs 6J–O, 9A–D). Axial area lanceolate (Fig. 6J–O), externally and internally faintly depressed with respect to virgae (Fig. 9A–D). Vimines short and wide (Fig. 9A, B). Striae composed of round to elliptic areolae, decreasing in size towards the axial area (Fig. 9A, B); wide elliptical areolae present toward valve face/mantle transition before and after the spine, sometimes accompanied by a narrower additional areola on valve mantle (Fig. 9E, F). Striae contained in a single depression in internal view (Fig. 9C, D). Well-developed volae, arising from the areolar inner periphery and projecting inwards (Fig. 9B–D). Flaps small and present on some valve face areolae (Fig. 9A, B), more commonly on larger mantle areolae (Fig. 9D–F). Spines originating from vimines at the valve face/mantle junction; solid, with round to elliptical base (Fig. 9A, B), as wide as the vimines they sit on (Fig. 9A, B, E); with cylindrical body and shallow concave sides (Fig. 9E, F), and spatulate tip with lateral projections and a serrate border pattern (Fig. 9D–F). Stipules incipient and subtending a small circular depression on the spine upper body (Fig. 9D, F). Apical pore fields covered by external flaps (Fig. 9A, E, F). Internally, apical pore field opening into roundish depression, revealing several rows of round poroids (Fig. 9D). Well-developed blister-like depositions present on abvalvar edge of mantle also covering both apices (Fig. 9D–F). Girdle elements variable in number, open, lacking pores, ligulated, with larger valvocopula (Fig. 9E, F).

Dimensions (n > 30): Length 6.7–35.6 μm; width 3.3–3.8 μm; striae 14–16 in 10 μm.

#### Etymology.

The species epithet alludes to the fact that this diatom has remained unidentified thus far and has been confused with morphologically similar taxa (see Discussion).

#### Distribution.

Found in the Sajama River.

### ﻿ Pseudostaurosira oblonga

Taxon classificationPlantaeFragilarialesStaurosiraceae

E. Morales, C.E. Wetzel & Ector sp. nov.

DE5D8FDE-E100-5905-B1C3-1C0AB9906AD5

Figs 6P–V (LM), 10A–F (SEM)

#### Holotype.

Slide BR-4680, Fig. 6R, Meise Botanic Garden, Belgium. **Isotype.** Slide DBOL-0249a, Diatomotheca Boliviensis (before HCUCB), Cochabamba, Bolivia.

#### Type locality.

Bolivia. Sajama Province, Department of Oruro, Sajama River, epipsammon, 17°30'33"S; 68°20'35"W, 4000 m elev., *leg.* G. Chávez, 05.07.2009.

#### Description.

**Figure 10.**
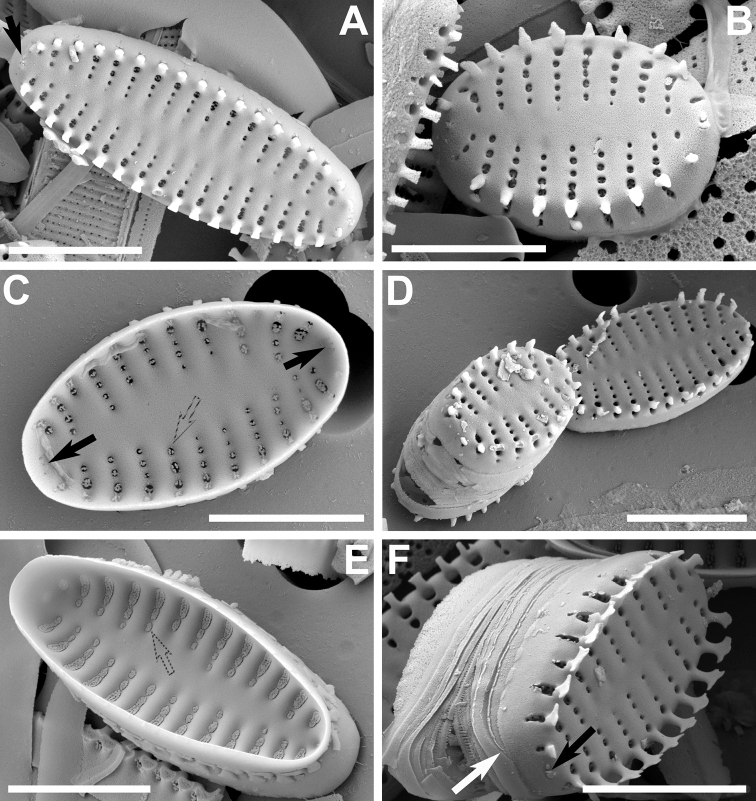
**A–F** SEM images of *Pseudostaurosiraoblonga* sp. nov. **A, B** external views showing apical pore fields covered with small flaps (black arrow in **A**) and other characteristics **C, E** internal valve features stressing on apical pore fields and striae in a depression (black arrows in **C** and dashed arrow in **C** and **E** respectively) **D, F** titled views of valves showing girdle bands (white arrow in **F**) and apical pore field (black arrow in **F**). Scale bars: 4 µm (**B, C, E, F**); 5 µm (**A, D**).

Frustules rectangular in girdle view, joined together by interlocking spines. Valves oblong, isopolar, with abrupt transition from valve face to mantle and broadly rounded apices (Figs 6P–V, 10A–F). Axial area lanceolate (Figs 6P–V), externally and internally depressed with respect to virgae (Figs 10A–F). Vimines short and wide (Fig. 10A–F). Striae composed of round to elliptic areolae, decreasing in size towards the axial area (Fig. 10A, B); wide trapezoid areolae present near the valve face/mantle transition at the base of the spine, sometimes accompanied by an additional narrower, round areola on valve mantle (Fig. 10A–F). Striae contained in a single depression in internal view (Fig. 10C, E). Developed volae, arising from the areolar inner periphery and projecting inwards (Fig. 10A, C, E). Flaps little-developed on valve face, developed on valve mantle, more commonly on larger mantle areolae (Fig. 10A, F). Spines originating from vimines at the valve face/mantle junction; solid, with elliptic base (Fig. 10F), as wide as the vimines they sit on (Fig. 10A, B, F); with a somewhat cylindrical body, concave sides, in the shape of a trapezium in side view (Fig. 10D, F), and widely spatulate tip with wide lateral projections (Fig. 10F). Stipules incipient or absent (Fig. 10A, D, F). Apical pore fields reduced, covered by small external flaps (Fig. 10A, B, F). Internally, apical pore field opening by means of a few very narrow, round poroids (Figs 10C). Small blister-like depositions present on abvalvar edge of mantle, absent from apices (Fig. 10A, D, F). Girdle elements variable in number, open, lacking pores, ligulated, with larger valvocopula (Fig. 10D, F).

Dimensions (n > 30): Length 6.9–13.5 μm; width 3.8–4.8 μm; striae 13–14 in 10 μm.

#### Etymology.

The species epithet refers to the widely ellipsoidal valve outline typical of this taxon.

#### Distribution.

Found in the Sajama River.

## ﻿Discussion

*Nanofrustulumcataractarum*, as seen in samples from the Desaguadero and Sajama rivers, is very similar to the type and other populations reported from around the world (Wetzel et al. 2013a; Grana et al. 2015; Beauger et al. 2019; Genkal 2021). The smaller dimensions of Bolivian specimens only expanded the initial measurements given by Hustedt (1938).

The more noticeable blisters on the mantle for Bolivian specimens could be due to the state of preservation of the material (more recent collection from Bolivia) and the possible higher availability of silica in the environment.

The lack of apical pore fields, stipules and flaps are typical in this taxon, but the most noticeable characteristic at the time of its identification under LM is the round shape of its valves and areolation pattern of the valve face and mantle, which resemble the smallest members of *Aulacoseira* Thwaites.

*Nanofrustulumrarissimum*, as discussed by Morales et al. (2019b), belongs in *Nanofrustulum* due to the quasifract nature of its girdle elements. The valvocopula in this case, however, is entire and ligulate. This difference with *N.cataractarum* that has all girdle elements quasifract has not been assessed in detail (Morales et al. 2019b), especially regarding the consequences for classification at the genus and species levels. It is known that other species can form morphologically different girdle elements (see the case of *Nitzschiatranstagensis* E. Morales, Novais, C.E. Wetzel, Morais & Ector in Morales et al. [2020, p. 34, figs 2–26], and other examples therein). A more detailed study of the variation of this character within the species currently assigned to *Nanofrustulum* is required.

*Pseudostaurosirasajamaensis* has large areolae proportional to its size (Morales et al. 2012b), which together with the gradual valve face/mantle transition, the sagittate-profiled spines with single or diapason-shaped tips, bearing serrate borders pointing downward are the main features to look for at the SEM level (Morales et al. 2012b, Fig. 2E, F, Table 1). Also characteristic at the latter level is the infrequent presence of a V-shaped cleft in the posterior side of the spine body. In the context of the taxa contrasted in Table 1, these are the features that are unique to this taxon.

**Table 1.** Comparison of *Pseudostaurosiraaedes* sp. nov., *P.pulchra* sp. nov and *P.sajamaensis* with other *Pseudostaurosira* and *Pseudostaurosiropsis* taxa of similar valve outline. Features in bold italic font are defining for each taxon.

**Feature/species**	***Pseudostaurosiraaedes* sp. nov.**	***P.altiplanensis* (Lange-Bertalot & Rumrich) E. Morales**	***P.pulchra* sp. nov.**	***P.sajamaensis* E. Morales & Ector**	***Pseudostaurosiropsisconnecticutensis* E. Morales**	***P.geocollegarum* (Witkowski) E. Morales**
**Valve dimensions (µm)**	L: 2.9–12.3 W: 2.1–2.6	L: 4.5–8.0 W: 2.8–3.6	L: 5.0–22.0 W: 2.4–3.0	L: 2–18 W: 2–4	L: 1.9–13.5 W: 1.5–4.7	L: 5–16 W: 2–4
**Stria density (in 10 µm)**	15	14–15	13–16	10–14	***17–20***	12–16
**Valve outline; axial area; virgae**	***Narrowly elliptic with rounded ends*** with abrupt transition of valve face to mantle; narrowly lanceolate; wide and slightly raised with respect to axial area, both elevated in internal view	***Broadly elliptic*** with abrupt transition of valve face to mantle; linear to narrowly lanceolate; ***wide and slightly raised together with axial area in both external and internal views***	***Narrowly lanceolate with rostrate to broadly rounded ends*** with abrupt transition of valve face to mantle; narrowly lanceolate; ***wide and raised with respect to axial area in external and internal view***	Narrowly elliptic in smaller specimens to elliptic with pointy ends in larger valves, ***with gradual transition from valve face to mantle***; widely lanceolate; wide and raised with respect to virgae in external view, both elevated in internal view	***Round to narrowly elliptic***; ***broadly elliptic***; wide, ***at the same level of axial area in external view, slightly raised in internal view***	***Lanceolate with subrostrate ends***; widely lanceolate; wide, raised with respect to axial area in external view, at the same level as axial area in internal view
**Areolae; volae; striae**	Narrow, elliptic to trapezoid; well-developed forming a tight mesh-like structure visible externally and internally; with 2, rarely 3 areolae, usually larger on valve mantle	***Wide***, ***often transapically elongated***; well-developed forming a loose mesh-like structure visible externally and internally; with 2 or more areolae of ***ca. the same size change away from the valve face/mantle junction***	Narrow, round to elliptic; well-developed forming a loose mesh-like structure visible externally and internally; typically composed by 2 areolae, usually larger on valve mantle	***Very wide***, round to trapezoid on valve face, ***trapezoid to elongate on mantle***; well-developed, forming a tight mesh-like structure as seen in internal view; usually composed of 2 areolae, wider ones on mantle, additional smaller ones more often seen on mantle	Narrow, round, of about the same size on valve face and mantle; absent, rotae present; typically composed of 2 areolae of similar size, additional areolae more frequent on mantle	Narrow, round, of about the same size on valve face and mantle; absent, rotae present; typically composed of 2 areolae of similar size, additional areolae more frequent on mantle
**Spines; stipules; flaps**	Solid, with elliptic to rectangular base, as wide as basal vimen, ***conical body, with a somewhat triangular profile***, with serrate and ***pointy tips***; well-developed, giving spines ***an arrowhead-like posterior profile***; well-developed, disk-like or bilobate on valve face, smaller, usually more than 2 on mantle	Solid, with elliptic base, ***narrower than basal vimen, cylindrical body with straight sides, spatulate tips with small and thin lateral projections***; ***very little developed***; incipient or little developed	Solid, round to elliptic base, ***wider than basal vimen***, ***flattened body with concave sides, straightly cut or slightly bifurcate apices***; absent; well-developed, circular on valve face, smaller, usually more than 2 on mantle	Solid, heavily silicified with elliptic base as wide as basal vimen, flattened body, sometimes ***with a V-shaped cleft in its posterior side, with diapason-shaped tips*** with serrate borders and ***pointy, downward, lateral projections***; well-developed, giving the ***spine a sagittate lateral profile***; well-developed, bilobate on valve face, less developed on mantle	Hollow, with elliptic base as wide as basal vimen, flattened, ***biconcave-spatulate body with bifurcate ends***; absent; absent	Hollow, round to elliptic base as wide as vimen, ***pyramidal as the body starts, becoming cylindrical toward the top***, with bifurcate ends; absent; absent
**Apical pore fields; mantle abvalvar blisters**	Present and very reduced, usually no more than 3 round poroids, ***opening in a single linear depression in internal view***; well-developed, covering the apical pore fields	Present or absent, composed of up to 3 rows of round poroids opening into a single, roundish internal depression; ***small***, covering the apical pore fields	Absent, well-developed, covering the apices	Absent or reduced, composed of a single row of round poroids opening in a single roundish depression in internal view; developed, present at apices but not covering the apical pore fields	Present or reduced, with small round poroids opening into a single roundish depression in internal view; small, present at apices but not covering apical pore fields	***Composed of up to three cavernous poroids, opening as large pores at the valve interior***; small, present at apices but not covering apical pore fields
**References**	This study	Rumrich et al. (2000), Seeligmann et al. (2018)	This study	Morales et al. (2012b), This study	Morales (2001), Beauger et al. 2019)	Morales (2001, 2002), Beauger et al. (2019), Radhakrishnan et al. (2020)

*Pseudostaurosirasajamaensis* is found in the same Desaguadero River sample together with *P.aedes* sp. nov. and *P.pulchra* sp. nov. However, at the LM level, the elliptic valves with pointy ends, the proportionately larger areolae, and the gradual transition between valve face and mantle in *P.sajamaensis* readily differentiate this taxon from the other two. The SEM features mentioned above, and which are defining for this taxon, can also be used to distinguish it from *P.altiplanensis* at this level (Lange-Bertalot & Rumrich) E. Morales (Rumrich et al. 2000, p. 220, pl. 14, figs 1–8; García et al. 2017, p. 112) (Table 1).

*Pseudostaurosiralinearis* (Pantocsek) E. Morales, Buczkó & Ector (in Morales et al. 2019b, p. 276, figs 3, 4) is another taxon with a gradual valve face/mantle transition, but it has very different features to those taxa included in Table 1. This taxon is fossil, and tends to produce longer valves; the axial area is at the same level as virgae in external and internal views, has spines with T-shaped tips and well-developed M or V-shaped stipules, and the apical pore fields are more- or less-developed with cavernous appearance externally and opening internally into a non-depressed area.

*Pseudostaurosirasajamaensis* has been recorded at the LM level from the Tunari Cordillera, in the Department of Cochabamba (E. Morales pers. obs.) located more than 200 km to the east of the Sajama and Desaguadero rivers. This cordillera is part of a long range that branches off the main Andes mountains, penetrating Bolivian territory and receiving the name of Eastern Cordillera (see “Study area” description in Morales 2020). These records, however, need to be confirmed with SEM. If confirmed, the range of this diatom would be extended to the eastern limits of the Bolivian Altiplano and their confluence with the Bolivian Dry Valleys.

*Pseudostaurosirapulchra* sp. nov. has the main distinguishing feature of *Pseudostaurosira*, the short and wide vimines (Morales et al. 2019b). Because the striae are mostly composed of two areolae, the vimines are mostly restricted to the valve face/mantle junction. The characteristics of the spines interrupting the striae, the areolae and associated structures (volae and flaps), the blister-like depositions and girdle elements are all in accordance with species currently ascribed to this genus.

Table 1 shows that the diagnostic features of *P.pulchra* sp. nov. are the narrow lanceolate valves with rostrate apices in larger specimens, becoming broadly rounded in smaller ones. Also the virgae wider than the striae, that are raised with respect to the axial area in internal and external views are typical in this species. Finally, the spines having a base that is wider than the vimines they sit on, and a body that is flattened with concave margins and has a flat top, or small bifurcate projections are also diagnostic in this taxon.

The taxon with the most similar morphology to *P.pulchra* sp. nov. is *Pseudostaurosiropsisgeocollegarum* (Witkowski & Lange-Bertalot) E. Morales (Witkowski et al. 1995, p. 734, figs 16–22; Morales 2002, p. 104, pl. 1, figs 1–9) (Table 1). At the LM level both have lanceolate valves, but in *P.pulchra* sp. nov. they are narrower (2.4–3.0 vs. 2–4 µm in *P.geocollegarum*), and the ends vary from rostrate to broadly rounded (subrostrate in *P.geocollegarum*). At the SEM level the most conspicuous difference is that *P.geocollegarum* does not possess volae and has rotae, a structure completely lacking in all *Pseudostaurosira*. *Pseudostaurosiropsisgeocollegarum* also has spines, the body of which starts with a pyramidal shape, becoming cylindrical toward the top, bearing bifurcate ends, a defining feature for the species (Table 1). Additionally, the apical pore fields in *P.geocollegarum* have a cavernous appearance with few pores that open as large isolated pores at the valve interior; this is also a defining feature for the species.

*Pseudostaurosirapulchra* sp. nov. has not been observed in other samples from the Altiplano and seems to be restricted to the Desaguadero and Sajama regions.

*Pseudostaurosiraaedes* sp. nov. has short and wide vimines, which place it in *Pseudostaurosira*. The vimines are mostly restricted to the valve face/mantle junction since the striae are commonly composed of only two areolae, one on the valve face and the other, usually larger, on the valve mantle. The other features such as spines located along the striae, the areolae and subareolar structures (volae and flaps), the blisters and girdle elements, are all in accordance with species currently placed in *Pseudostaurosira*.

Despite the apparent difficulty in the distinction of this diatom from other similar taxa (Table 1), it has distinctive features that set it apart from them. The narrowly elliptic shape with rounded ends, the arrowhead-like spines (configuration given by the well-developed stipules) with a conical body and serrate tips, and the apical pore fields composed of only a few round poroids and opening internally into a single linear depression are all diagnostic features of this species.

At the LM level, the most similar taxon to *P.aedes* sp. nov. is *Pseudostaurosiropsisconnecticutensis* E. Morales (2001, p. 117, figs 7a-l) (Table 1). Especially for smaller specimens, both present elliptic valves but the stria density is much higher in *P.connecticutensis* (17–20 vs. 15). Also, the spines in the latter are flattened with a biconcave-spatulate body and bifurcate ends. As a member of *Pseudostaurosiropsis*, *P.connecticutensis* has rotae and lacks volae. Additionally, this taxon has virgae that are at the same level of the axial area in internal view, while they are slightly raised in internal view. Spines with a flattened, spatulate body, concave on the sides, and with bifurcate ends. These characteristics of the virgae with respect to the axial area, and the spines are defining features for *P.connecticutensis*.

Smaller representatives of *P.aedes* sp. nov. can also resemble *Pseudostaurosiraaltiplanensis* (Lange-Bertalot & Rumrich) E. Morales. At the LM level, however, *P.altiplanensis* is much wider (2.8–3.6 vs. 2.1–2.6 in *P.aedes* sp. nov.), the valves are broadly elliptic instead of narrowly elliptic with rounded ends and the striae are long, composed of transapically very elongate areolae. At the SEM level, the virgae are wide and slightly raised together with axial area in both external and internal views; the spines are narrower than vimines, with a cylindrical body with straight sides, spatulate tips with small and thin lateral projections, bearing little-developed stipules. The blisters on the abvalvar side of the mantle are comparatively smaller. All these SEM features are defining characters for *P.altiplanensis* (Table 1).

*Pseudostaurosiraaedes* sp. nov. was only found in the Desaguadero River in the present study, but it was illustrated before by Rumrich et al. (2000, pl. 13, fig. 26, only figure that is not numbered in the plate) and identified as “*Staurosirabrevistriata* Grunow” (*Pseudostaurosirabrevistriata* (Grunow) D.M. Williams & Round, 1987, p. 276; for figures of type material refer to Morales et al. 2015, figs 107–143 since figures in Williams & Round and incorrectly labeled). The valve shape, the features of the areolae, spines and blisters are all confluent with what is described here as *P.aedes* sp. nov.

*Pseudostaurosiraushkaniensis* Kulikovskiy & Lange-Bertalot (in Kulikovskiy et al. 2015, p. 28, pl. 16, figs 1–12, pl. 17, figs 1–8) resembles both *P.pulchra* sp. nov. and *P.aedes* sp. nov. under LM. However, the mantle areolae in *P.ushkanenis*, being large and occupying almost the entire shallow, curved mantle in a pervalvar direction, are clearly visible showing two rows of these structures per stria in valve view. At the SEM level, the spines are located on virgae, have a cylindrical body and a spatulate tip. The apical pore fields are well-developed with neatly arranged rows of poroids in external view and a depressed elliptic plate with clearly round poroids in internal view. All of these features differ from the two Bolivian taxa (see them in Table 1).

*Pseudostaurosiraheteropolaris* sp. nov. has wide and short vimines, a character that places it in *Pseudostaurosira*. This species is distinguished by its short, ovoid to elliptic, heteropolar valves, the wide base of the volae which give the areolae a C-shape appearance, the pinnatifid profuse bifurcations of the spine tips and the small blisters on the abvalvar edge of the mantle (Table 2).

The evident heteropolar configuration of *P.heteropolaris* sp. nov. is shared with *P.clavatum* E. Morales (2002, p. 107, pl. 1, figs. 22–34, pl. 4, figs 1–6), but this is a very different species with larger valves (8–20 µm), coarser striae (11–12), wide, elliptic to trapezoid areolae on valve face and mantle (one on each), profusely bifurcate volae, flat, hollow spines with serrated borders and two ligulae projected laterally, and well-developed apical pore fields on both valve apices.

Another species with evident heteropolar shape is *P.conus* Kulikovskiy & Lange-Bertalot (in Kulikovskiy et al. 2015, p. 26, pl. 15, figs 15–18). At the LM level the valves are clavate with rostrate, sometimes subcapitate head pole and a very fine foot pole. External views of this taxon under SEM are unknown, but from the single figure presented by Kulikovskiy et al. 2015 it can be seen that the spines are small and conical and that the apical pore fields are well-developed and composed of several rows of poroids, features absent in *P.heteropolaris* sp. nov.

The same authors presented *P.gomphonematoidea* Kulikovskiy & Lange-Bertalot (in Kulikovskiy et al. 2015, p. 26, pl. 19, figs 1–3), another clavate taxon with a broadly rounded head pole and a thinner foot pole. The areolae are round to elliptical and the volae are not as developed as in *P.heteropolaris* sp. nov. The spines are completely flat and the apical pore fields are well-developed, features not present in the new taxon from Bolivia.

As evident in Table 2, *P.heteropolaris* sp. nov. is different from morphologically similar species in the genus, from which it can be distinguished in a first instance under LM by the features cited above, especially by the small size and heteropolar valves. The rest of the diagnostic features need to be revealed by SEM. Among the latter, the features of the areolae and spines completely separate this taxon from the others in the present manuscript.

**Table 2.** Comparison of *Pseudostaurosiraheteropolaris* sp. nov and *P.oblonga* sp. nov. with morphologically similar, congeneric species. Features in bold italic font are defining for each taxon.

**Feature/species**	***P.alvareziae* Cejudo-Figueiras, E. Morales & Ector**	***P.americana* E. Morales**	***P.bardii* Beauger, C.E. Wetzel & Ector**	***P.heteropolaris* sp. nov.**	***P.oblonga* sp. nov.**
**Valve dimensions (µm)**	L: 10–18 W: 3.6–5.0	L: 6.0–38.0 W: 4.5–5.0	L: 4.0–6.5 W: 3.0–5.5	L: ***3.0–4.3*** W: 2.6–3.3	L: 6.9–13.5 W: 3.8–4.8
**Stria density (in 10 µm)**	13–15	16–18	12–16	13–16	13–14
**Valve outline; axial area; virgae**	Elliptic with faintly subrostrate to broadly rounded apices, isopolar; narrowly lanceolate, faintly depressed in external view with respect to virgae, at the same level as virgae in internal view; much wider than striae	Lanceolate with cuneate apices, isopolar; linear, ***at same level as virgae in external and internal view***; wider than striae	Elliptic to round with broadly rounded ends, isopolar; lanceolate to elliptic, depressed with respect to virgae in external view, at the same level as virgae in internal view; wider than striae	***Ovoid*** (sometimes elliptic in small specimens), ***heteropolar***; elliptic, externally slightly depressed with respect to virgae, internally at the same level as the latter; much wider than striae	***Oblong*** with widely rounded apices, isopolar; lanceolate, ***depressed in external and internal view with respect to virgae***; wider than striae
**Areolae; volae; striae**	Wide, round to elliptic; well-developed and forming a tight mesh-like structure seen in external and internal view; composed of up to 6 areolae decreasing in size away from valve face/mantle junction, ***larger areolae contiguous to spines on valve face and mantle***	Wide, round to elliptic; well-developed and forming a tight mesh-like structure visible externally and internally; composed of up to 7 areolae ***with little size variation away from valve face/mantle junction on valve face***, valve mantle with larger areolae varying from elliptic to trapezoid	Wide, round to elliptic; developed and directed toward valve interior; composed of up to 5 areolae decreasing in size away from valve face/mantle transition, first areola on mantle trapezoid and as large as first areola on valve face	Narrow, elliptic to round or hemispherical at the axial area; well-developed and directed toward valve interior, ***base of volae wide giving areolae a C-shape***; composed by up to 7 areolae decreasing in size away from valve face/mantle transition, first areola on mantle as large as first areola on valve face	Narrow, round to elliptic; well-developed forming a tight mesh-like structure visible externally and internally; composed by up to 6 areolae decreasing in size away from valve face/mantle transition, first areola on mantle trapezoid and larger
**Spines; stipules; flaps**	Solid, elliptic base, as wide as basal vimen, flattened body and widely spatulate tips with broad lateral projections; small, ***conical***; incipient on valve face, developed on valve mantle	Solid, elliptic base, as wide as basal vimen, cylindrical body with a ***V-shaped middle opening*** and openly concave sides, widely spatulate tips with serrate borders; ***well-developed, almost covering entire first areola on mantle***; well-developed, more common on valve mantle	Solid, elliptic base, wider than basal vimen, flattened body, ***triangular in side view***, spatulate tips ***with serrate borders***; developed; developed but more frequent on mantle areolae	Solid, elliptic to rectangular base, wider than basal vimen, cylindrical body with concave sides, spatulate tips ***with pinnatifid bifurcations***; absent; absent	Solid, elliptic base, as wide as basal vimen, quasi-cylindrical body with concave sides, ***trapezium-shaped in side view***, spatulate tips with wide lateral projections; ***incipient*** or absent; little developed on valve face, larger on valve mantle
**Apical pore fields; mantle abvalvar blisters**	Well-developed, externally with ***wide round poroids and covered by contorted flaps***; small, absent from apices	Well-developed, of cavernous appearance externally, ***poroids lie at bottom of troughs***, internally round poroids open into a shallow depression; well-developed, present at apices	Very reduced almost completely externally covered by flaps, only a pair of poroids can be seen, of cavernous appearance, internally only 3 narrow, round poroids can be seen, which open into a shallow depression; developed and present at apices	Very reduced, externally up to 3 cavernous poroids; ***small present at apices***	Reduced, covered by small external flaps, internally opening through few, narrow, ***unsunk poroids***; small absent from apices
**References**	Cejudo-Figueiras et al. (2011)	Cejudo-Figueiras et al. (2011), Morales et al. (2013b)	Beauger et al. (2019)	This study	This study

*Pseudostaurosiravulpina* stat. nov. has a triradiate form, the first key feature to its identification under LM. This combined with the swellings mid-way between arms most surely give a positive identification. Confirmation at the SEM level is given by the apical pore fields, somewhat depressed into the three valve apices and opening to the valve interior by a single non-depressed porous plate. In Table 3, we also annotate that the externally depressed axial area with respect to the virgae, and internally at the same level as the latter is an exclusive feature of *P.vulpina* among triradiate forms. Within the latter, *P.vulpina* is also the only one possessing small conical spines.

**Table 3.** Comparison of *P.vulpina* stat. nov. with most similar triradiate species in *Pseudostaurosira* and *Pseudostaurosiropsis* that have LM and SEM information available. ND=not determined. Features in bold italic font are defining for each taxon.

**Feature/species**	***P.iztaccihuatlii* V.H. Salinas & D. Mora**	***P.vulpina* stat. nov.**	***Pseudostaurosiropsistriradiatum* (E. Morales) Kulikovskiy, Glushchenko & B. Karthick**
**Valve dimensions (µm)**	L: 6.2–10.8 W: ND	L: 4.8–13.0 W: 4.1–5.6	L: 7–10 W: ND
**Stria density (in 10 µm)**	12–18	14–16	14–16
**Valve outline; axial area; virgae**	Triradiate; irregularly triangular, externally and internally depressed with respect to the virgae; wide	Triradiate; irregularly triangular and ***externally depressed with respect to the virgae, internally at the same level as the latter***; wide	Triradiate; ***triangular, with concave sides***, depressed with respect to the virgae in external and internal view; ***much wider than striae***
**Areolae; volae; striae**	Roundish to transapically elongate; well-developed and ***with ring-like depositions distorted in different ways at the valve interior***; composed of several areolae, a single one on the valve mantle	Roundish to transapically elongated; well-developed and ***with inverted cone-like accumulations of different shape at the valve interior***; composed of several areolae, a single one on the valve mantle	***Circular***; absent, ***with rotae instead***; composed of up to 4 areolae 2 on valve face and 2 on the mantle
**Spines; stipules; flaps**	***Large, conical base, spatulate tip***, slightly slender than basal vimen; absent; absent	***Small, conical***, slender than basal vimen, ***sometimes shapeless and occurring on virgae and vimines***; absent; absent	***Small, conical, wider that basal vimen***; absent; absent
**Apical pore fields; mantle abvalvar blisters**	Depressed, not known from valve interior view; large and extending to the apical portions but below the apical pore field	Depressed, opening into a non-depressed porous plate at the valve interior; large and extending to the apical portions but below the apical pore field	***Not depressed, opening internally into a circular depressed zone***; ND
**References**	Salinas et al. (2020)	Rumrich et al. (2000), this study	Morales (2005)

Depositions in the internal surface of the striae have also been reported in *Pseudostaurosiradecipiens* E. Morales, G. Chávez & Ector (in Morales et al. 2012b, p. 44, figs 2–10, 39–44). However in *P.decipiens* there are two superimposed disks on each areolar opening, while in *P.vulpina* there is only one, inverted cone-like structure, hollow at the center or with extra siliceous deposition in its interior (Fig. 7F). These cones seem to appear as material accumulates over the copiously branched volae. Since the areolae are elongated in *P.vulpina*, the base of the cones is distorted, assuming a somewhat triangular or ovoid configuration (Fig. 7F).

*P.vulpina* as presented in Rumrich et al. (2000) lacks the internal cone-like depositions, but this seems to be due to erosion on the valves since the volae seem to have also been lost to a great extent from the areolae. These cone-like depositions are also found in the recently described fossil *Pseudostaurosiracrateri* Marquardt & C.E. Wetzel (in Marquardt et al. 2021, p. 107, figs 1–57). However, this latter taxon is very different from the triradiate *P.vulpina* in that it has a lanceolate shape, very narrow striae with more areolae on the valve mantle than on the valve face, which results in a wide axial area, depressed externally and internally, and apical pore fields in internal view reminiscent of *P.parasitica* (W. Smith) E. Morales (2003a, p. 287, figs 27–43, 54–54, 60, 64), i.e. an elevated plate that contains several round poroids. Externally, these apical pore fields resemble those in *P.vulpina*, although they are much larger proportionately to the valve size in *P.crateri*.

*Pseudostaurosiraiztaccihuatlii* V.H. Salinas & D. Mora (in Salinas et al. 2020, p. 196, figs 18–35) is most probably conspecific with *P.vulpina*. The only two differences between the population from Mexico and the Andean population are the larger spines interrupting the striae of the former and the shape of the internal striae depositions (Table 3, features that have not been traditionally used for separation of species within the genus). The structure of the areolae and volae structure is similar in both taxa and there is no external or internal velum, as misinterpreted by Salinas et al. (2020); if a velum were present, *P.iztaccihuatlii* would have to be placed in a different genus. Both the structure of the areolae and volae are extensively used to separate *Pseudostaurosira* from other genera and to separate its species, as we have done herein. The internal striae depositions are ring-like in the latter and cone-like in *P.vulpina*.

Also from Table 3, it is clear that *Pseudostaurosiropsistriradiatum* (E. Morales) Kulikovskiy, Glushchenko & B. Karthick (Morales 2005, p. 129, Figs 74–79, 127–132; in Radhakrishnan et al. 2020, p. 173) is different from *P.vulpina* due to its axial area in the shape of a triangle with concave sides, the much wider virgae than striae, the circular areolae with rotae, small conical spines wider than vimines, and the apical pore fields that are not depressed exteriorly, but they do internally.

A third taxon that could be compared with *P.vulpina* is *Staurosiramercedes* Lange-Bertalot & Rumrich (in Rumrich et al. 2000, p. 224, pl. 10, figs 12–14), a taxon that had been introduced under the name Fragilariabrevistriatavar.trigona Lange-Bertalot nom. inval. without a diagnosis in Krammer & Lange-Bertalot (1991a, pl. 117, fig. 7B). No SEM images of *S.mercedes* have been published, but at the LM level the valves are triangular with concave sides (boomerang-like) and the ends are cuneate rather than rostrate as they are in *P.vulpina*. Lange-Bertalot also introduced the name Staurosirapseudoconstruensvar.trigona (in Rumrich et al. 2000, pl. 15, figs 1, 2), but this name was not accompanied by a diagnosis either. The LM figure the authors presented (fig. 2) seems to be a teratological form and has a similar general appearance in the lower side of the triradiate shape as a medium-sized valve of *P.vulpina*. The SEM image presented in Rumrich et al. (2000, pl. 15, fig. 1) confirms this. The shape of the areolae, the position of spines and the sunken apical pore fields in the close-up image are all similar to *P.vulpina*. We also note that Metzeltin & Lange-Bertalot (1998, pl. 2, fig. 5) presented a valve of *P.vulpina* (judging by valve shape and features of striae, areolae, spines and apical pore fields) that they identified as “Fragilariabrevistriata(Grunow s. lato)var.trigona Lange-Bertalot” a name that appears only in this text and without a formal description.

The change in status of *P.vulpina* is here justified by the finding of a population with mixed frustule sizes, a probable indication that the species is reproducing asexually and sexually, independently from the nominate variety, *P.laucensis*, and growing isolated from it in the Desaguadero River.

This taxon has been reported and illustrated from the Argentinian (Tchilinguirian et al. 2018), Bolivian (Morales et al. 2007) and Chilean Altiplano (Rumrich et al. 2000), with possible records from Europe still to be confirmed by SEM. It is possible that it was also identified with other names. For example, Servant-Vildary (1978, p. 3, pl. 2, fig. 13, see also LM image in Servant-Vildary 1986, pl. 2, fig. 27) presented a drawing that closely resembles our Figs 6C and D, with triangular shape and inflated sides and mammillate ends, which the author named “Fragilariaconstruens(Ehr)Grunvar.exigua (W. Smith) Schuls[sic]” (=Staurosiraconstruensvar.exigua (Ehrenberg) H. Kobayasi (in Mayama et al. 2002, p. 90, for illustrations refer to Krammer and Lange-Bertalot 1991a, pl. 117, figs 4–7 (LM) and Potapova 2014, p. 80, fig. 98 (SEM, under “Staurosiraconstruensf.exigua”)). From the latter references the var.exigua is distinguished by subcapitate ends, incipient spines developing on virgae, and the unbroken striation pattern, since the striae are formed by small, apically elongate areolae. Additionally, the apical pore fields are not sunken and contain several rows of neatly arranged poroids.

*Pseudostaurosirafrankenae* sp nov. is an additional cruciform species included in the genus (Table 4). It has the main feature of species currently assigned to it, namely the wide and short vimines, but it also shares with them the features of the areolae, spines and flaps, apical pore fields and girdle elements.

**Table 4.** Comparison of *Pseudostaurosirafrankenae* sp. nov. with selected, similar, congeneric, and cruciform to broadly lanceolate taxa. Features in bold italic font are defining for each taxon. *Internal view of *P.caballeroae* is unknown.

**Feature/species**	***P.australopatagonica* M.L. García, L.A. Villacís, Maidana & E. Morales**	*P.caballeroae* V.H. Salinas, D. Mora, R. Jahn & N. Abarca*	***P.decipiens* E. Morales, G. Chávez & Ector**	***P.frankenae* sp. nov.**	***P.laucensis* Lange-Bertalot & Rumrich**	***P.parasitica* (W. Smith) E. Morales**	***P.pseudoconstruens* (Marciniak) D.M. Williams & Round**
**Valve dimensions (µm)**	L: 20–25 W: 6.5–9.0	L: 13.8–17.7 W: 6.9–8.5	L: 4–29 W: 4–6	L: 8.7–12.0 W: 6.7–7.7	L: 5.5–20.0 W: 3.5–5.5	L: 9–18 W: 4.5–5.0	L: 4–22 W: 3–7
**Stria density (in 10 µm)**	10–12	13–14	13–15	14	14–15	19–21	15–18
**Valve outline; axial area; virgae**	Cruciform to rhomboid with subcapitate apices; lanceolate, wider at central area, clearly depressed with respect to virgae in outer view, slightly depressed in internal view; much wider than striae	Cruciform with narrowly rounded ends; lanceolate, wider at central area; clearly depressed with respect to striae in external view; wide	Lanceolate with rostrate ends; lanceolate wider at central area, externally slightly depressed with respect to virgae, internally at the same level as the latter; wide	Cruciform with broadly rounded ends; lanceolate, wider at central area; clearly depressed with respect to striae in external and internal view; ***slender than striae***	Lanceolate to rhomboid with narrowly subrostrate to cuneate ends; lanceolate, faintly depressed with respect to virgae in outer view, flat in internal view; wide	Lanceolate with narrowly rounded to subcapitate ends; lanceolate, wider at central area, clearly depressed with respect to virgae in external and internal view; ***as wide as striae***	Cruciform with broadly rounded to subcapitate ends; lanceolate wider at central area, slightly depressed with respect to virgae in external and internal view; much wider than striae
**Areolae; volae; striae**	Round at apices to elliptically elongate; large generally growing opposite from shorter axis of areolae, ***internally forming a dendritic pattern***; composed of 1–2 areolae on valve face, 1 large, round to ovoid on valve mantle	Round at apices to elliptically elongate, sometimes only 1 very long on valve face; smaller, bifurcate and growing from longer areolar axis; composed of 1–2 areolae on valve face, valve mantle areola not clearly illustrated	Round to ovoid; ***diapason shaped*** and further bifurcate at the valve interior, allowing ***the internal deposition of two concentric disks of inorganic material***; composed of 1–2 areolae on valve face and 1 large, trapezoid on mantle, sometimes an extra round one present on mantle	Round to elliptic; smaller, bifurcate, allowing the internal ***deposition of an elliptic disk of inorganic material***; composed of 1–4 areolae on valve face and a single, large, trapezoid one on mantle	Round to elliptic; developed ***with ring-like depositions distorted in different ways at the valve interior***; composed of 1–2 areolae on valve face, decreasing in size toward axial area and a single, slightly larger, elliptic to trapezoid on the mantle	Round at apices to elliptically elongate; small, usually originating from smaller axis of valve; composed typically of 1, unusually 2, areolae on valve face, ***typically one smaller areola on valve mantle***	Round to elliptic; smaller, bifurcate, originating from inner areolar perimeter; composed of 1–4 areolae on valve face, 1–2 on valve mantle, ***of same size as valve face areolae***
**Spines; stipules; flaps**	***Incipient, forming a short arch-like structure*** extending from virgae to virgae; ***absent; absent***	***Very thin, flattened***, extending from virgae to virgae, ***forming an undulate to dentate pattern on valve face/mantle transition***; absent; absent	Solid, elliptic base, as wide as basal vimen, flattened body with concave sides, spatulate or heart-shaped tip; absent; absent, ***only mineral depositions resembling floating disks on outer areolar opening***	Solid, round to elliptic base, wider than basal vimen, ***triangular in side view***, flattened upper body with bifurcate tip; absent; ***disk-like, 1 persistent in valve face areolae; 1–3 in mantle areolae***	***Incipient and occurring as whitish depositions along valve face/mantle transition***; absent, absent	Absent; absent; absent	Solid, ***with long elliptic base, shorter than basal virgae on which they grow***, flattened body with concave sides, ***highly branched tips; absent; absent***
**Apical pore fields; mantle abvalvar blisters**	Cavernous and large, almost covering entire valve apex with poroids at the base of troughs, internally opening into a single elliptic depression; small, absent from the apices	Cavernous and large, almost covering entire valve apex with poroids at the base of troughs, internally unknown; excess depositions impede visualization in original illustrations	Cavernous, from 1 to several rows of poroids can be seen externally, internally a roundish depression contains several rows or poroids; developed, present including at valve apices	Cavernous, only one transapical row of poroids can be seen externally, internally, a round depression contains various rows of poroids; developed, present including at valve apices	***Non-cavernous***, sunken onto valve apex in external view, internal view unknown; small, not covering apices	***Cavernous, visibly sunken onto valve apex*** and occupying almost its entirety, pores lie at bottom of troughs, ***internally plaque of pores is raised***; ***very small*** and absent from apices	***Very reduced externally***, internally opening into a small depressed circular area; small and present at apices
**References**	García et al. (2021)	Salinas et al. (2020)	Morales et al. (2012b)	This study	Servant-Vildary (1986), Rumrich et al. (2000)	Morales (2003a, 2010)	Williams and Round (1987)

This new species has several distinguishing features that set it apart from other congeneric taxa with cruciform valve outline. The virgae are slender than striae, internally the striae bear a single elliptic, occluding disk (a character unique in the entire genus), Spines have a triangular basal configuration when viewed from the side; the areolae bear persistent flaps (Table 4). Additionally, the apical pore fields are covered by a siliceous deposition that only reveals a single row of poroids. We have not studied variation of this latter feature, it seems to be constant in the species but it requires confirmation.

Due to their cruciform shape, *P.australopatagonica* M.L. García, L.A. Villacís, Maidana & E. Morales (in García et al. 2021, p. 3, figs 2–35), *P.caballeroae* V.H. Salinas, D. Mora, R. Jahn & N. Abarca (2020, p. 199, figs 36–50) and *P.pseudoconstruens* (Marciniak) D.M. Williams & Round (1987, p. 277, figs 28–31 mistakenly under “*Pseudostaurosirabrevistriata*”) are the closest morphological relatives to *P.frankenae* sp. nov. However, from Table 4, it can be seen that *P.australopatagonica* has the volae forming an internal dendritic pattern, which is a character unique for the species. Additionally, this latter species has incipient spines forming arched, convex structures on the vimines, interrupting the striae at the valve face/mantle junction. The species from Argentina lacks stipules as *P.frankenae* sp. nov. does, but the latter has flaps, which are absent in *P.australopatagonica*.

In the case of *P.caballeroae*, the spines are thin and flat, forming an undulate dentate pattern over vimines (where most of the spine base and lower body lie) and virgae. While this species and *P.frankenae* sp. nov. lack stipules, the latter has flaps, which are lacking in *P.caballeroae*.

*Pseudostaurosirapseudoconstruens* has the closest overall valve shape to *P.frankenae* sp. nov. However, *P.pseudoconstruens* has the spines on the virgae and not on vimines as the rest of the species discussed in the present work (Williams and Round 1987). The base of those spines is shorter and they possess highly branched tips. Both stipules and flaps are lacking in this species. Additionally, the apical pore fields are highly reduced and are not cavernous as in *P.frankenae* sp. nov.

*Pseudostaurosiradecipiens* E. Morales, G. Chávez & Ector (in Morales et al. 2012b, p. 44, figs 2–11, 39–44), *P.laucensis* and *P.parasitica* (W. Smith) E. Morales (2003a, p. 287, figs 27–43, 54–58, 60, 64) have a lanceolate valve shape, this being the first distinguishing feature to separate them from *P.frankenae* sp. nov.

From Table 4, it is worth noticing that *P.laucensis*, a taxon that can be distinguished from morphological related species by its incipient spines occurring on virgae and vimines along the entire valve face/mantle transition, its internal ring-like depositions on the striae and its non-cavernous apical pore fields, had already been shown by Servant-Vildary (1986, pl. 3, figs 31b–36), identified as “*Fragilariabrevistriata* Grunow”. Both references show populations with the same valve features, although the latter reference shows a valve interior with the typical ring-like depositions on the striae. These depositions are unique to *P.laucensis* among taxa with cruciate to lanceolate valves. The ring-like configuration, however, is shared with the triradiate *P.iztaccihuatlii* (see Table 3 and discussion above). These two taxa also share the apical pore field configuration and the difference in areolar width between valve face and valve mantle areolae.

Regarding the incipient spines in *P.laucensis*, these also occur in *P.vulpina* as shown in Fig. 7E, but also in Rumrich et al. (2000, pl. 10, figs 8, 10, 11). Both taxa also share the apical pore field configuration.

*Pseudostaurosiraocculta* sp. nov. is distinguished from similar species under LM by its lanceolate shape with subrostrate, somewhat square and broadly rounded apices (Table 5). In this latter table it can be seen that there are several distinguishing features typical of this new species at the SEM level. From these, the circular depression on the spine body, subtended by an incipient stipule, stands out. Also, the flap coverings, twisted, almost externally completely obstructing the apical pore fields are characteristic in this taxon. The remaining species in Table 5 have their own features that separate them from their morphologically close relatives. Valve dimensions are not very useful to differentiate these species and, apart from valve shape, SEM features should be used to distinguish them.

**Table 5.** Comparison of *Pseudostaurosiraocculta* sp. nov. with morphologically similar species within the genus. Features in bold italic font are defining for each taxon.

**Feature/species**	***P.polonica* (Witak & Lange-Bertalot) E. Morales & M.B. Edlund**	***P.occulta* sp. nov.**	***P.oliveraiana* Grana, E. Morales, Maidana & Ector**	***P.subsalina* (Hustedt) E. Morales**	***P.zolitschkae* M.L. García, S. Bustos, Maidana & E. Morales**
**Valve dimensions (µm)**	L: 8–23 W: 3–4	L: 6.7–35.6 W: 3.3–3.8	L: 19.0–39.5 W: 3.5–5.5	L: 10–36 W: 4.0–5.3	L: 8.5–28.0 W: 3.5–5.0
**Stria density (in 10 µm)**	13–15	14–16	11–17	13–14	11–14
**Valve outline; axial area; virgae**	***Broadly elliptic, rarely clavate***, broadly rounded apices, larger specimens slightly constricted in the middle; lanceolate, at same level as virgae in both external and internal views; wider than striae	Lanceolate with subrostrate, ***squarish***, broadly rounded apices; lanceolate, faintly depressed with respect to virgae in external and internal view; wider than striae	Lanceolate with ***subcapitate to cuneate ends***; broadly lanceolate, at same level as virgae in both external and internal views; wider than striae	Lanceolate ***with parallel sides*** and subrostrate apices; linear to narrowly lanceolate, faintly depressed with respect to virgae in external and internal view; wider than striae	Lanceolate with subrostrate apices, ***smaller valves biconvex with cuneate, pointy ends***; broadly lanceolate, ***at the same level as virgae in external view, slightly depressed in internal view***; much wider than striae
**Areolae; volae; striae**	***Wide***, elliptic; well-developed, originating from longer areolar axis, and forming a tight mesh in internal view; rarely formed by more than 3 areolae, larger on valve	Narrow, round to elliptic; developed, originating from the inner areolar periphery, forming a tight mesh-like structure visible internally; with up to 4 areolae on valve face and up to 2 on valve mantle, ***usually larger near valve face/mantle transition***	Narrow, elliptic to trapezoid; developed, originating from longer axis of areolae; with up to 3 areolae, larger on valve face	Narrow, round to elliptic; ***poorly developed***, originating from inner areolar periphery and projecting inwards; with up to 5 areolae, decreasing in size towards the axial area, ***a single larger areolae present on valve mantle***	Narrow, elliptic to ***8-shaped***, trapezoid on valve mantle; developed and forming a mesh-like pattern in inner view; ***with 2 areolae of about the same size on face and mantle***
**Spines; stipules; flaps**	Hollow, with elliptic base, narrower than basal vimen, flattened body ***with very openly concave sides, spatulate tips with somewhat straight bifurcate projections***; present but poorly developed; poorly-developed on valve face and mantle	Solid, with elliptic to rectangular base, as wide as basal vimen, ***cylindrical body with open concave sides***, with spatulate tips that bear two lateral projections and ***serrate borders***; incipient, ***subtending a circular depression on spine body***; poorly developed on valve face, ***well-developed on valve mantle, spatulate***	Solid, round to elliptic base, as wide as basal vimen, flattened body and ***tips with an overall inverted trumpet shape***, ***tips bifurcate once or twice***; absent; absent	Solid, round to elliptic base, as wide as basal vimen, flattened body with somewhat straight but flaring sides and ***spatulate tip bearing two small lateral projections*** with serrate borders, ***with an overall ice cream cone shape***; incipient; poorly developed	Hollow, with round to elliptic base, narrower ***than basal vimen***, with flattened body and ***typically T-shaped tips***; ***well-developed and lobed***; ***developed on valve mantle, lobed***
**Apical pore fields; mantle abvalvar blisters**	Somewhat developed, with a few, ***large, round pores, sunken into an irregular depression at the valve apex***, internally opening into a small roundish depression; well-developed, apparently not present at apices	***Developed, externally covered by contorted flaps in external view***, internally opening in a single ca. elliptic depression, revealing several rows of round poroids; well-developed, also present at valve apices	Somewhat developed, ***externally sunken into an elliptic depression at the valve apex***, internally opening into a ca. ***elliptic depression with an elevated central area***, and showing several round poroids; developed but absent from apices	Somewhat developed, ***sitting on a step-like transition between valve face and mantle***, externally large roundish pores lie in an irregular depression, internally, the narrow, round pores open into a single roundish depression; developed but present at apices	***Small, externally reduced to large pores with cavernous appearance***, internally, small round pores lie in a roundish depression; developed, absent from apices
**References**	Morales & Edlund (2003)	This study	Grana et al. (2018)	Cejudo-Figueiras et al. (2011)	García et al. (2021)

*Pseudostaurosirasubsalina* (Hustedt) E. Morales (in Cejudo-Figueiras et al. 2011, p. 69, figs 2–33, 94–99, 107, 109, 111) is perhaps the closest species to *P.occulta* sp. nov. at the morphological level (Table 5). However, the former taxon has valves with parallel sides, poorly-developed volae and a single valve mantle areola, larger than the rest of areolae along the same stria. In the case of spines, these have an overall flared shape, somewhat resembling an ice cream cone, spatulate body and tip, the latter bearing two small lateral projections. The apical pore fields in *P.subsalina* sit on a step-like apex.

Likewise, the remaining species in Table 5 can be readily separated from *P.occulta* sp. nov. For each taxon we highlight the salient distinguishing features. *Pseudostaurosirapolonica* (Witak & Lange-Bertalot) E. Morales & M.B. Edlund (2003, p. 235, figs 25–32, 45–50) has broadly elliptic valves, sometimes clavate, the areolae are very wide, and the spines are hollow. *Pseudostaurosiraoliveraiana* Grana, E. Morales, Maidana & Ector (in Grana et al. 2018, p. 63, figs 2–15, 16–24) has valves with subcapitate to cuneate ends, trumpet shaped spines, and externally sunken apical pore fields that open interiorly into an elliptical depression but with a raised central area. In turn, *P.zolitschkae* M.L. García, S. Bustos, Maidana & E. Morales (in García et al. 2021, p. 11, figs 81–91, 103–109) has the widest range of morphological variability of all taxa included in Table 5 producing smaller valves with clearly biconvex sides and pointy ends. The areolae acquire an 8-shape configuration due to the thick origin of the volae that arise from the longer (transapical) axes of the areolae. The spines in this taxon are T-shaped and the apical pore fields are cavernous and small.

*Pseudostaurosiralinearis* has a similar overall shape to *P.occulta* sp. nov. However, as stated above when comparing it to *P.sajamaensis*, *P.linearis* is a fossil species, and it has much wider areolae, has more coarsely striated valves (12–14 striae in 10 m versus 14–16 in *P.occulta* sp. nov.) and has all the other features cited in the comparison to *P.sajamaensis* that *P.occulta* sp. nov. does not possess.

*Pseudostaurosiraocculta* sp. nov. has been reported before under the name “*Fragillariazeilleri* Heribaud[sic]” by Servant-Vildary and Roux (1990, p. 276, fig. 29). The shape of the valve imaged under SEM, the characteristics of the areolae and spines resemble what we are describing here as the new species *P.occulta*. Type material of *Fragilariazeilleri*, now *Pseudostaurosirazeilleri* (Héribaud) D.M Williams & Round (1987, p. 276, no figures), was studied by Serieysol (1988), who showed a taxon with widely elliptic valves and cuneate ends, axial area varying from linear to elliptic, striae composed of narrower areolae, usually one larger after the spine, on the valve mantle. The spines in this taxon have an overall flat, trumpet-like shape without lateral projections or serrate borders. The apical pore fields in *P.zeilleri* are clearly visible, are somewhat cavernous but lack any external coverings.

*Pseudostaurosiraoblonga* sp. nov. can be distinguished by clearly oblong valve shape, the externally and internally depressed axial area, the trapezium-shaped profile of the spines and the incipient stipules that appear to be facultative (Table 2). This species is clearly different from *P.heteropolaris* sp. nov. simply based on the small size of the latter and its heteropolar valve shape, although we present other distinguishing features in Table 2.

The remaining species differ from *P.oblonga* sp. nov in the following salient features, selected from Table 2. *Pseudostaurosiraalvareziae* Cejudo-Figueiras, E. Morales & Ector (in Cejudo-Figueiras et al. (2011), p. 69, figs 34–73, 100–105, 106, 108, 110) characteristically has two larger areolae before and after the spines along the same stria, the stipules are small and conical and the apical pore fields are typically covered by twisted flaps. *Pseudostaurosiraamericana* E. Morales (in Cejudo-Figueiras et al. 2011, p. 70, figs 74–93, 112–115. See also Morales et al. 2013b) has a V-shaped middle opening in the spine body, large stipules covering the subtending areolae on the valve mantle, and an apical pore field in which the external openings of the poroids lie in troughs. *Pseudostaurosirabardii* Beauger, C.E. Wetzel & Ector (in Beauger et al. 2019, p. 4, figs 2–56), on its part, has spines with a triangular profile, spines that have tips with serrate borders.

As it has been seen here, defining *Pseudostaurosira* as a genus distinguished by short (apical extension) and wide (transapical extension) vimines (relative to the size of the areolae) (Morales et al. 2019b), allows the analysis of the variability of other features for the discrimination of species. That is, there is a large chance that the features of the vimines result in an evolutionary character that defines and separates the genus from others; however, this possibility for vimines to be synapomorphic requires demonstration, and we will perform the necessary cladistic analyses once a fair amount of species have been described and the type material of remaining key species has been studied (e.g. *P.pseudoconstruens*, *P.microstriata* (Marciniack) Flower [2005, p. 65], etc.).

As expressed in the descriptions of the new species and the comparative tables presented herein, the salient features that can be used to distinguish species are the features of the axial area, virga and vimines, the areolae and subareolar features (volae, rotae, flaps and internal depositions), the spines (base, body and tips) and stipules, and apical pore fields. We have tried to find differences in other features such as valve shape, morphometric measurements, stria density, blisters and girdle bands, but we have been unsuccessful in finding sufficient variability across a large number of species. As more species are described and type material is re-analyzed, it is possible that the latter characters take more importance in defining species.

### ﻿A note on our morphologically based approach to “araphid” diatom taxonomy

Biodiversity conservation is a crucial endeavor in the face of climate change, pollution and habitat loss (Prathapan and Rajan 2020). It is already recognized that to carry out this conservation process “a good and constantly updated taxonomical knowledge is fundamental” (Khuroo et al. 2007). The problem in South America, as in many parts of the world, is that the taxonomic impediment (Wilson 1985; Wheeler et al. 2004) is even more vexing since very few universities and research groups in the continent are actively trying to solve it (perhaps not true, at least in some countries, for insects, fish and higher plants, as argued by de Carvalho et al. 2014a). And this lack of attention currently happens in a region grouping several countries declared as biodiversity hotspots and high-biodiversity wilderness areas (IUCN - International Union for Conservation of Nature 2013), but also as the least caring about nature and the environment. For example, Brazil, Bolivia, Peru and Colombia (in that order) are among the top 10 countries with the highest loss of primary forest in the world (Weisse and Goldman 2021).

In Bolivia, habitat loss is a deeply preoccupying problem since there is a lack of strong environmental policies and even the Government itself constantly breaks the existing law in order to expand the agricultural frontier, exploit oil, minerals, timber, etc. (Castro et al. 2014). The situation of the aquatic systems in Bolivia, those associated with urban development or even those in protected areas that are now being damned, is also worrying (U.S. Army Corps of Engineers 2004). The recent loss of Lake Poopó to the mining industry and contamination is one of the largest recent environmental catastrophes in South America and an example of the degree of degradation of aquatic resources in the country (Richard and Contreras 2015). It is evident, therefore, that there is a tremendous need for discovering, describing, identifying and cataloguing the diversity present in the affected areas in order to provide a historic record of what is (was) present in these sites for conservation and restoration purposes. In particular, the Sajama and Desaguadero regions are currently being affected by mining and urbanization of some of their areas, though the effects of both have not yet been officially reported.

Although there is a growing body of literature, the main diatom treatises for the region have been conducted by foreign authors (e.g. Frenguelli 1939; Servant-Vildary 1986; Rumrich et al. 2000) and not always reflecting topographic, bioclimatic and ecosystem variability, resulting in an incomplete account that often manifests in skewed conclusions regarding the richness and composition of diatom communities in high and lowlands (see discussions in Morales et al. 2012b, 2014c, 2020). For the existing literature, besides the shortcomings in sampling and geographic coverage, at least for small “araphids”, there is a history of taxonomic drift, misidentification and a severe lack of pictorial support for floristic surveys in different regions of the continent (Morales et al. 2014c; García et al. 2021). Misidentification and poor illustration are problems that we have also shown here in the case of *P.vulpina* appearing in the literature under Fragilariabrevistriatavar.trigona nom. inval. (Krammer and Lange-Bertalot 1991a) and Fragilariaconstruensvar.exigua (Servant-Vildary 1978, 1986). Other examples are those of *P.occulta* sp. nov. lumped under the name *P.zeilleri* (Servant-Vildary and Roux 1990), and *P.laucensis* being mistaken for *P.brevistriata* (Servant-Vildary 1986).

Whether this diatom biodiversity account should be done using molecular or morphological approaches is (for now and given the urgency to document as much of that diversity as possible in a short time) a matter of availability of funds and equipment, which are scant in the country. Currently, the cheapest route to diatom biodiversity reporting is to concatenate LM and SEM approaches via international collaboration.

But besides the reality of research conditions in the country, there is also the more general matter of whether morphological or molecular taxonomy should be used in the urgent endeavor to solve the biodiversity crisis (Wilson 1985). What we have done here is to produce hypotheses on distinctiveness based on morphological characters, by comparison among morphologically closely related species, breaking down features that are currently underexplored in “araphid” diatoms. This breakdown produces a substantial amount of information, as seen in Tables 1–5, that could later be used to support barcoding and/or DNA data, which in turn can be used to test the hypotheses we raised here.

The ongoing debate on whether molecular or morphological data should prevail over another has revealed important pros and cons of both approaches (Savolainen et al. 2005; Evans et al. 2007; Pires and Marinoni 2010; Zimmermann et al. 2011). But as implied by Lipscomb et al. (2003), Teletchea (2010), and Kahlert et al. (2019), it is much more productive to think of a fusion of both approaches than to think that either of them, in isolation, could produce a reliable identification system or even fairly approximate the actual number of species present in nature. For diatoms, a first attempt to concatenate morphological and molecular datasets has been tried already as in the case of the marine epizoic *Tursiocola* spp. (Frankovich et al. 2018), and in the case of freshwater “araphids” in the genus *Fragilaria* (Kahlert et al. 2019), although a uniform protocol for the treatment of both datasets and consensus trees fusing molecular and morphological data have not yet been achieved. But the latter is not surprising since nowadays very little has been done in terms of translating molecular data into functional perspectives of the diatom phenotype (i.e. we know very little about what genes produce which characters, a process that could be beneficial for the establishment of homologous traits and recognition of diagnostic features (see e.g. Cox 2010)), although considerable progress, albeit still weak regarding the molecular connection, is expressed by Hale and Mitchell (2001) and Aitken et al. (2016). Even now, it is interesting to note that current molecular analyses, outstandingly exemplified by the construction of barcode databases, is undoubtedly a type of morphological analysis, i.e. the analysis of the morphology of the DNA molecule.

These and other shortcomings highlighted by Morales et al. (2019b) have determined that an integrative taxonomy (Pires and Marinoni 2010) in diatom research is still not in clear sight and that much more work is still required to produce reliable and practical accounts of the biodiversity of these organisms. Kahlert et al. (2019) also point out historic shortcomings of morphological data, referring to the lack of uniformity in morphological descriptions of taxa, the fact that old descriptions are based on LM, and that it is not always possible to observe all diagnostic features during routine analyses. We have been trying to solve these issues pointed by Kahlert et al. (2019) for the past decade-and-a half. Through the revision of type material, we have attempted the documentation of traditional and new diagnostic features, expanding original descriptions and confirming or re-ascribing taxa into newly erected genera (such as those in Williams and Round 1987). We have also discovered new taxa, described and documented them following the current standards (Morales 2001, 2002, 2003a, b, 2005, 2006; Morales and Edlund 2003; Edlund et al. 2006; Morales and Manoylov 2006a, b; Morales et al. 2005, 2009, 2010a, b, c, 2012a, b, 2013a, 2014a, b, 2015, 2019a, b, 2021; Siver et al. 2006; Cejudo-Figueiras et al. 2011; Wetzel et al. 2013a, b; Rioual et al. 2014; Talgatti et al. 2014; Van de Vijver et al. 2014, 2020a, b; Almeida et al. 2015, 2016, 2017; Grana et al. 2015, 2018; Wetzel and Ector 2015, 2021; Wengrat et al. 2016; García et al. 2017, 2021; Beauger et al. 2019; Seeligmann et al. 2018; Guerrero et al. 2019; Marquardt et al. 2021). The amalgamation of LM and SEM has been crucial in our work, even though type materials were not always in a good state of preservation.

The growing amount of morphological and re-analyzed historical data, and the relative easiness and low cost of the methods employed in their collection, continue to be a convenient way to contribute data for the study of biodiversity (e.g. elaboration of species inventories, numbers and distribution, morphological variation), ecology (e.g. autecology, assemblages and their relations to their environment, biogeography) and applied fields such as biostratigraphy, paleoecology, bioindication, bioprospection, climate change research and preservation/conservation/recuperation practices). Therefore, the resolution of historic taxonomic entanglements, description of new species and clarification of taxonomic boundaries based on morphological analyses continue to be valid and they are a very much needed practice.

Regarding the standardization of terminology and the format of the descriptions, we have been putting forward expanded diagnoses of taxa (e.g. descriptions provided herein) which, although they tend to be repetitive in the case of shared features among taxa, constitute a deep account of as many observable features under LM and SEM as it has been possible for us to collect. Also, regarding the provision of good comparative analyses, we have provided tables contrasting key diagnostic features, that we are herein expanding even further to include previously underexplored characters (Tables 1–5, see also Table 1 in Morales et al. 2019b for a comparison of small “araphids” at the genus level).

The revisionary work and study of type material we have been doing, which result in the morphological redefinition of taxa boundaries, is not only descriptive work (Haszprunar 2011). Descriptions, by means of accurate, standard terminology, and concatenation of LM and SEM are valuable records that constitute the taxonomic history of an entity. Thorough descriptions do not only reveal the original author´s intentions and appreciation of the importance of certain characters, but they also offer guidance to subsequent interpretation in the context of what was known about morphology and key characters of taxa at the time of the first description of a taxon.

These revisionary activities and the results we have achieved over the years for the small “araphid” diatoms provide concrete evidence that much more work is still needed to describe in morphological terms the diversity of these diatoms and that this process is completely justified given the current needs and the state of the art in diatom diversity studies (Mann and Vanormelingen 2013). Meanwhile, molecular studies continue their parallel advancement, not without problems similar to those encountered by morphologists (Bailet et al. 2020), nevertheless augmenting the chance that in the near future we will be able to produce a more complete molecule-phenotype system that allows building a natural compendium of “araphid” diatom biodiversity, perhaps even meeting the goals of the Grand Linnean Enterprise (Wheeler et al. 2012; Prathapan and Rajan 2020). But, a compendium must also be translated into a classification system that reflects evolutionary history (de Carvalho et al. 2014b), a systematic approach that is of outmost importance in sustainable conservation practices for populations and communities are not static groupings, but rather they have evolutionary trajectories in the context of their environment, which are important to consider for their preservation, conservation and recuperation (Olivieri et al. 2015).

In the context of the paleolimnological research done in the Bolivian Altiplano on “araphid” diatoms, and in the face of the taxonomic inconsistencies encountered in some publications, paleolimnological data must be reviewed, but for this to take place there must be a fair knowledge of the current biodiversity of the group. These can be accomplished by wider surveys than the one we presented here, based only on two sites and referencing a few others. As discussed by Morales (2020) under-representativeness and under-sampling of a rather varied geographical landscape are serious flaws in the knowledge of Andean diatoms. Thus, much work needs to be devoted to better represent the composition of the diatom flora present in this region. Our effort expressed here and in Morales et al. (2012b) only represents the first steps to unravel the diatom community developing at these localities. As explained by the authors, the sample from Desaguadero contains more than 200 species with restricted distribution and more than half are unknown taxa in multiple genera, not only “araphids” (see also discussion in Morales et al. 2014c). Thus, wider surveys may yield a high number of new taxa completely changing the current view of the Andean diatoms as being dominated by cosmopolitan taxa, but based on sampling of easily accessible, human-influenced areas (Rumrich et al. 2000).

## Supplementary Material

XML Treatment for Nanofrustulum cataractarum

XML Treatment for Nanofrustulum rarissimum

XML Treatment for Pseudostaurosira sajamaensis

XML Treatment for Pseudostaurosira pulchra

XML Treatment for Pseudostaurosira aedes

XML Treatment for Pseudostaurosira heteropolaris

XML Treatment for Pseudostaurosira vulpina

XML Treatment for Pseudostaurosira frankenae

XML Treatment for Pseudostaurosira occulta

XML Treatment for Pseudostaurosira oblonga
